# IL-34 affects fibroblast-like synoviocyte proliferation, apoptosis and function by regulating IL-17

**DOI:** 10.1038/s41598-021-95839-1

**Published:** 2021-08-12

**Authors:** Xin Li, Yimeng Lei, Ziyu Gao, Gang Wu, Wei Gao, Liping Xia, Jing Lu, Hui Shen

**Affiliations:** 1 Department of Rheumatology, 1st Affiliated Hospital of Jin Zhou Medical University, Jin Zhou, 121000 China; 2 Department of Rheumatology, 1st Hospital of China Medical University, Shen Yang, 110001 China; 3 104k Class 86, China Medical University, Shen Yang, 110001 China; 4 Department of General Surgery, 1st Affiliated Hospital of Jin Zhou Medical University, Jin Zhou, 121000 China

**Keywords:** Rheumatoid arthritis, Cytokines, Inflammation

## Abstract

Rheumatoid arthritis (RA) is a chronic inflammatory disease characterized by proliferation and insufficient apoptosis of fibroblast-like synoviocytes (FLSs).The biology and functions of interleukin (IL)-34 are only beginning to be uncovered. We previously demonstrated IL-34 could upregulate the expression of IL-17 in RA patients. In this study, the 3-(4,5-dimethylthiazol-2-yl)-2,5-diphenyltetrazolium bromide (MTT) assay and flow cytometry of Annexin V and PI staining were performed to assess cell proliferation and apoptosis progression in RA-FLSs after stimulated with increasing concentrations of IL-34, respectively. Inflammatory cytokines and angiogenic factors were measured using quantitative real-time PCR, Western blotting and ELISA. We explored the association between IL-34 and RA-FLS proliferation and apoptosis in the context of RA. Stimulating RA-FLSs with different concentrations of IL-34 significantly promoted the proliferation and inhibited the apoptosis of RA-FLSs in a concentration-dependent manner. Neutralization of IL-17 with the IL-17 inhibitor plumbagin (PB) reduced the effects of IL-34. Proinflammatory cytokine (IL-17A IL-6 and tumor necrosis factor-α, TNF-α) and angiogenic factor (vascular endothelial growth factor, VEGF and hypoxia-inducible factor-1α, HIF-1α) expression was markedly upregulated in RA-FLSs stimulated by IL-34. PB-mediated inhibition of IL-17A also decreased the expression of IL-6, TNF-α, HIF-1α and VEGF in RA-FLSs. Taken together, these findings suggest that targeting IL-34 production in RA-FLSs may be a therapeutic strategy for RA.

## Introduction

Rheumatoid arthritis (RA) is a complex autoimmune disease characterized by symmetrical inflammation of synovial joints. Abnormal inflammation and immune response are the main clinical manifestations in RA patients, but the etiology and pathogenesis are still unknown^1^. Cytokines play key roles in driving synovial cell activation, which leads to joint damage. Interleukin (IL)-34 was recently discovered through functional screening of the extracellular proteome. IL-34 is widely expressed in the brain, liver, spleen, heart and other tissues^2^. Recent studies have shown that IL-34 is expressed in fibroblast-like synoviocytes (FLSs), subsynovium and the intima in RA patients. IL-34 expression was significantly correlated with the severity of synovitis^3,4^. Compared with those of the healthy population and patients with osteoarthritis, the levels of IL-34 in FLSs, serum and synovial fluid of RA patients were significantly increased^5–7^, and the level of IL-34 in synovial fluid was positively correlated with the total white blood cell count^4^. In addition, in RA patients, serum IL-34 levels are positively correlated with the titer of rheumatoid factor (RF) and anti-cyclic citrullinated peptide antibodies (ACPAs)^8^. The IL-34 level in the synovial fluid of RA patients with high disease activity (DAS 28 ≥ 3.2) was significantly higher than that of patients with low disease activity (DAS 28 < 3.2)^9^. Thus, significantly elevated IL-34 levels may be a valid marker of RA activity. However, the specific mechanism of IL-34 in RA is still unclear.

Th17 cells can secrete the cytokine IL-17^10^. IL-17 can promote joint local inflammation and cause the release of a large number of inflammatory cytokines, such as TNF-α, IL-6 and IL-1. These inflammatory cytokines indirectly promote the expression of receptor activator of NF-κB ligand (RANKL) in synovial cells or osteoclasts and cause osteoclast precursor cells to differentiate into osteoclasts^11^. IL-17 is highly expressed in synovial tissues and the synovial fluid of RA patients. IL-17 expression is closely associated with the degree of RA joint damage^12^.

In the pathogenesis of RA, angiogenesis can lead to pannus formation in the synovial membrane, causing synovial erosion and bone destruction. Moreover, the degree of vasculogenesis in the synovium is closely associated with disease recurrence and joint destruction^13^. Therefore, angiogenesis is one of the important factors leading to the loss of joint function and disability in RA patients. Under inflammatory stimulation, the RA-FLSs can secrete a variety of angiogenic factors, including vascular endothelial growth factor (VEGF) and hypoxia-inducible factor (HIF)^14–16^. Other factors are also involved in vasculogenesis in the RA synovium, such as angiopoietin (Ang)1 and its tyrosine kinase receptor Tie2^14,17^ monocyte chemoattractant protein (MCP)-1, IL-8, IL-17, and interferon-inducible protein (IP) 10. The association between IL-34 and angiogenesis in RA has not been clearly reported. In this study, the effects of exogenous recombinant human IL-34 on the secretion and expression of inflammatory cytokines and angiogenesis-related factors in FLSs from RA patients were observed. Furthermore, we applied IL-17 inhibitors to observe whether IL-34 affects the function of RA-FLSs by regulating the expression of IL-17.

## Materials and methods

### Reagents

Recombinant IL-34 was purchased from R&D Systems (Minneapolis, USA). The IL-17 inhibitor plumbagin (PB) was purchased from Novus (Centennial, USA). PB (10 mg)was slowly dissolved in 530 µl of anhydrousdimethyl sulfoxide (DMSO) by gentle vortexing and administered to cells in media containing the solvent at a final concentration of 4 µM. A cell proliferation and cytotoxicity assaykit was purchased from Biyuntian Reagent Company (Nantong, China). A FITC Annexin V-FITC/PI apoptosis detection kit was purchased from BD Biosciences (New York, USA). Dulbecco’s modified Eagle’s medium (DMEM) and 10% fetal bovine serum (FBS) were purchased from HyClone (Utah, USA).

### RA-FLS culture

Human RA-FLSs(MH7A cells) were purchased from Hongshun Biotechnology Co., Ltd. (Shanghai, China). All cells were cultured in DMEM supplemented with 10% FBS in an incubator with 5% CO_2_ at 37 °C. When the FLSs had grown into a firmly adherent cell monolayer with 80% confluence, the cells were digested with 0.25% trypsin (Logan, USA) to generate a single-cell suspension. The cells were passaged at a 1:2 ratio.

### Cell proliferation assay

The effect of IL-34 on FLS proliferation was measured by the 3-(4,5-dimethylthiazol-2-yl)-2,5-diphenyltetrazolium bromide (MTT) assay. Cells were plated in 96‑well plates at a density of 5000 cells/well overnight, after which they were treated with different concentrations of IL-34 (0, 25, 50, and 100 ng/ml) or IL-34 (100 ng/ml) plus PB (4 µM). After being incubated for 48 h at 37 °C in a humidified incubator, MTT (5 mg/ml in PBS) was added to each well and incubated for 4 h. Subsequently, the medium was removed, and 0.1 ml of buffered formalin was added to each well. The absorbance was recorded on a microplate reader at a wavelength of 570 nm. Three independent replicates were performed. A proliferation curve was generated with the concentration of IL-34 on the horizontal axis and the OD value on the vertical axis.

### Quantification of apoptosis

For apoptosis analysis, 1 × 10^5^ FLSs/well were plated on six-well plates, treated with different concentrations of IL-34 (0, 25, 50 and 100 ng/ml) or IL-34 (100 ng/ml) plus PB (4 µM) for 48 h and then harvested. The cells were labeled with annexin V and PI. Apoptosis rates were determined by flow cytometry (BD Biosciences) and analyzed using FlowJo software (version 7.6.1). Three independent replicates were performed. The percentage of apoptosis was calculated by counting the numbers of annexin V‑positive and PI-positive cells.

### Reverse transcription-PCR (RT-PCR)

RA-FLSs were seeded in 6-well plates at a density of 3 × 10^5^ cells/well and cultured with different concentrations of IL-34 (0, 25, 50 and 100 ng/ml) or IL-34 (100 ng/ml) plus PB (4 µM) for 48 h. Total RNA was obtained from FLSs using TRIzol reagent (Invitrogen; Thermo Fisher Scientific, Inc.). A 0.5-μg sample of total RNA was reverse transcribed using the PrimeScript RT Master Kit. The resulting cDNA was used for amplifcation by RT-PCR with the SYBR Premix Ex TaqTM Kit and ABI Prism 7000 (Applied Biosystems, Norwalk, CT). The conditions were as follows: initial denaturation at 95 °C for 10 min, followed by 40 cycles of 95 °C for 5 min, 60 °C for 30 s and 72 °C for 30 s. Each sample was analyzed in triplicate. At the end of the PCR cycles, melting curve analysis was performed to validate the specific generation of each expected PCR product. The primer sequences used are summarized in Table 1. The expression levels of different genes were calculated using the 2^−△△Ct^ method^18^. Three independent replicates were performed.

**Table 1:** List of the sequence of gene primers.

Gene	Forward primer (5′-3′)	Reverse primer (3′-5′)
IL-17A	CTCTGTGATCTGGGAGGCAAA	CTCTTGCTGGATGGGGACA
TNF-α	CTGCCTGCTGCACTTTGGAG	ACATGGGCTACAGGCTTGTCACT
IL-6	AAGCCAGAGCTGTGCAGATGAGTA	TGTCCTGCAGCCACTGGTTC
VEGF	GAGCCTTGCCTTGCTGCTCTA	CACCAGGGTCTCGATTGGATG
HIF-1α	CAGCCGCTGGAGACACAATC	TTTCAGCGGTGGGTAATGGA
β-actin	CATGTACGTTGCTATCCAGGC	CTCCTTAATGTCACGCACGAT

### Western blot analysis

RA-FLSs were seeded in 6-well plates at a density of 3 × 10^5^ cells/well and treated with different concentrations of IL-34 (0, 25, 50 and 100 ng/ml) or IL-34 (100 ng/ml) plus PB (4 µM) for 48 h. Total protein was extracted from RA-FLSs on ice using RIPA lysis buffer (R0010, Solarbio, Beijing) supplemented with 0.1% phenylmethylsulfonyl fluoride (PMSF) according to the manufacturer’s protocol. The isolated proteins were obtained after centrifugation at 14,000×*g* for 10 min. Equivalent amounts of protein (40 μg) from each sample were separated by SDS-PAGE and transferred to polyvinylidene fluoride (PVDF) membranes (00010, Millipore Co., USA). The PVDF membranes were blocked with 5% BSA for 2 h. Then, the membranes were incubated with primary antibodies overnight at 4 °C on a shaker. The primary antibodies used were as follows: anti-IL-6 (ab6672, Abcam, England, 1:1000), anti-TNF-α (ab9635, Abcam, England, 1:1000), anti-IL-17 (ab79056, Abcam, England, 1:1000), anti-VEGF (ab52917, Abcam, England, 1:1000), and anti-HIF-1α (ab51608, Abcam, England, 1:250). Then, the membranes were incubated with a horseradish peroxidase-conjugated goat anti-rabbit secondary antibody (ab205718, Abcam, England, 1:1000) for 2 h. After the membranes were washed with TBST3 times, chemiluminescence (ECL) was performed, and a gel imaging apparatus (Bio-Rad, ChemiDoc MP, USA) and analysis software (Image Lab Software) were used. Three independent replicates were performed (Supplementary Information).

### Cytokine secretion analysis by enzyme-linked immunosorbent assay (ELISA)

RA-FLSs were seeded in 96-well plates at a density of 4 × 10^3^ cells/well and treated with different concentrations of IL-34 (0, 25, 50 and 100 ng/ml)or IL-34 (100 ng/ml) plus PB (4 µM) for 48 h. RA-FLSs were treated for 48 h and collected, and for the levels of proinflammatory cytokines (IL-6, IL-17, TNF-α) and angiogenic factors (VEGF,HIF-1α) were analyzed with ELISA kits (R&D Systems, USA) as recommended by the manufacturer. Melting curve analysis was performed to validate the specific generation of each expected PCR product. The primer sequences used are summarized in Table 1. The expression levels of different genes were calculated using the 2^−△△Ct^ method^18^. Three independent replicates were performed for each index.

### Statistical analysis

GraphPad Prism (Version 6.01) statistical software was used for statistical analyses. The data are presented as the mean ± standard error (SEM). The differences between four groups were tested by one-way analysis of variance (ANOVA). The differences between two groups were tested by two-tailed Student’s t-tests. Differences were considered statistically significant when *p* < 0.05.

## Results

### IL-34 promoted the proliferation of RA-FLSs by regulating IL-17

**Figure 1:**
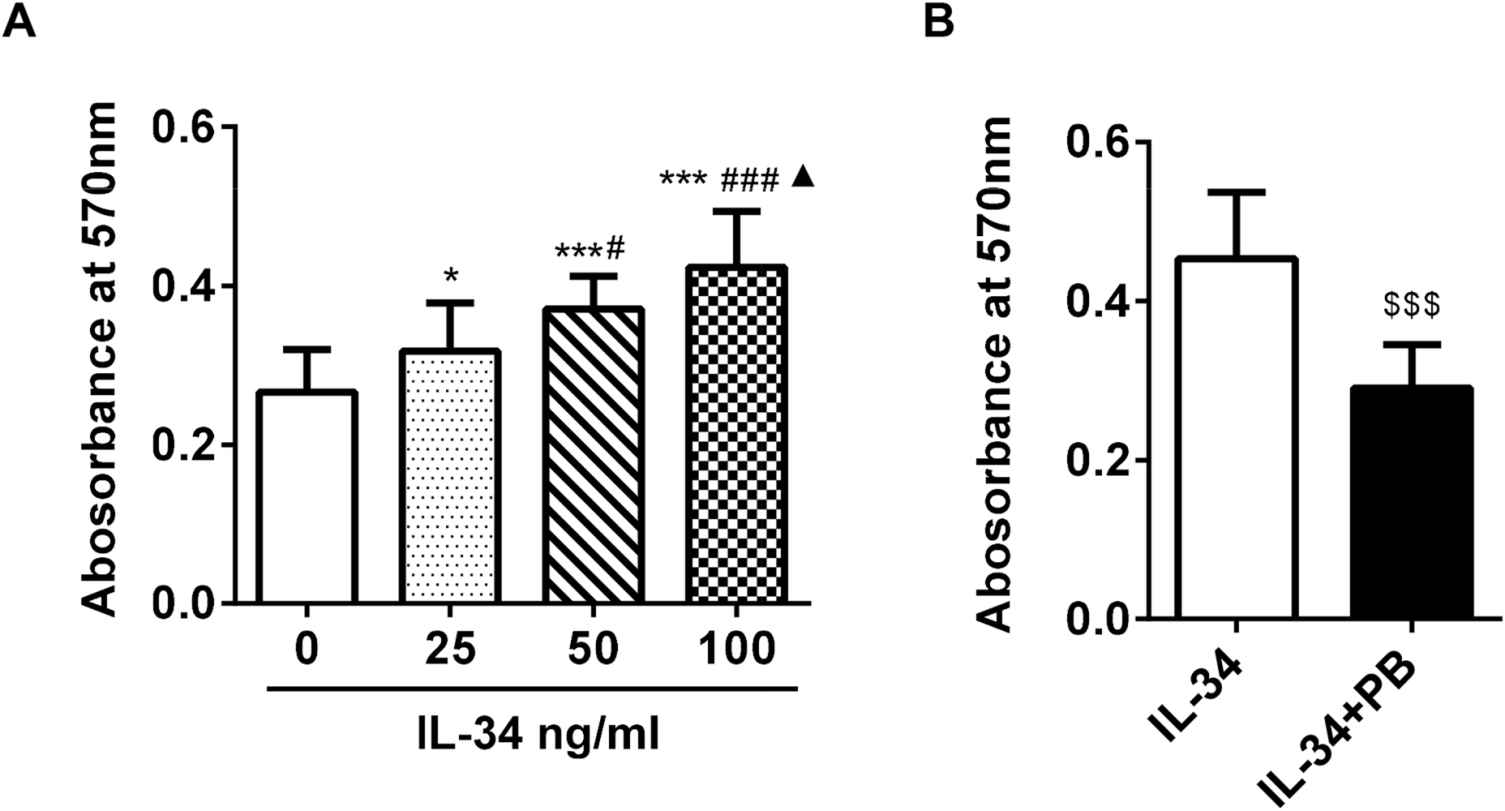
IL-34 promoted the proliferation of RA-FLSs by regulating IL-17. (**A**) The MTT assay was used to measure RA-FLS proliferation. RA-FLSs were treated with different concentrations of IL-34 (0 ng/ml, 25 ng/ml, 50 ng/ml and 100 ng/ml). (**B**) RA-FLSs were treated with IL-34 (100 ng/ml) or IL-34 (100 ng/ml) + PB (4 µM).The differences between the four groups were tested by ANOVA. Statistical comparisons between two groups were performed by t-tests. The data are expressed as the mean ± SEM. *P < 0.05, ***P < 0.001 vs the 0 group; ^#^*P* < 0.05, ^###^*P* < 0.001 vs the 25 group; ^▲^*P* < 0.05 vs the 50 group; ^$$$^*P* < 0.001 vs the IL-34 group.

To determine whether IL-34 promotes RA-FLS proliferation, MTT assays were performed. We found that RA-FLS proliferation was greatly enhanced by stimulation with different concentrations of IL-34 (0, 25, 50 and 100 ng/ml) (Fig. 1A). However, when we used plumbagin (PB, an IL-17A inhibitor), the effect of IL-34 was decreased (Fig. 1B). These results indicate that IL-34 promotes growth, and this effect may be diminished by an IL-17A inhibitor.

### IL-34 inhibited RA-FLS apoptosis by regulating IL-17

**Figure 2:**
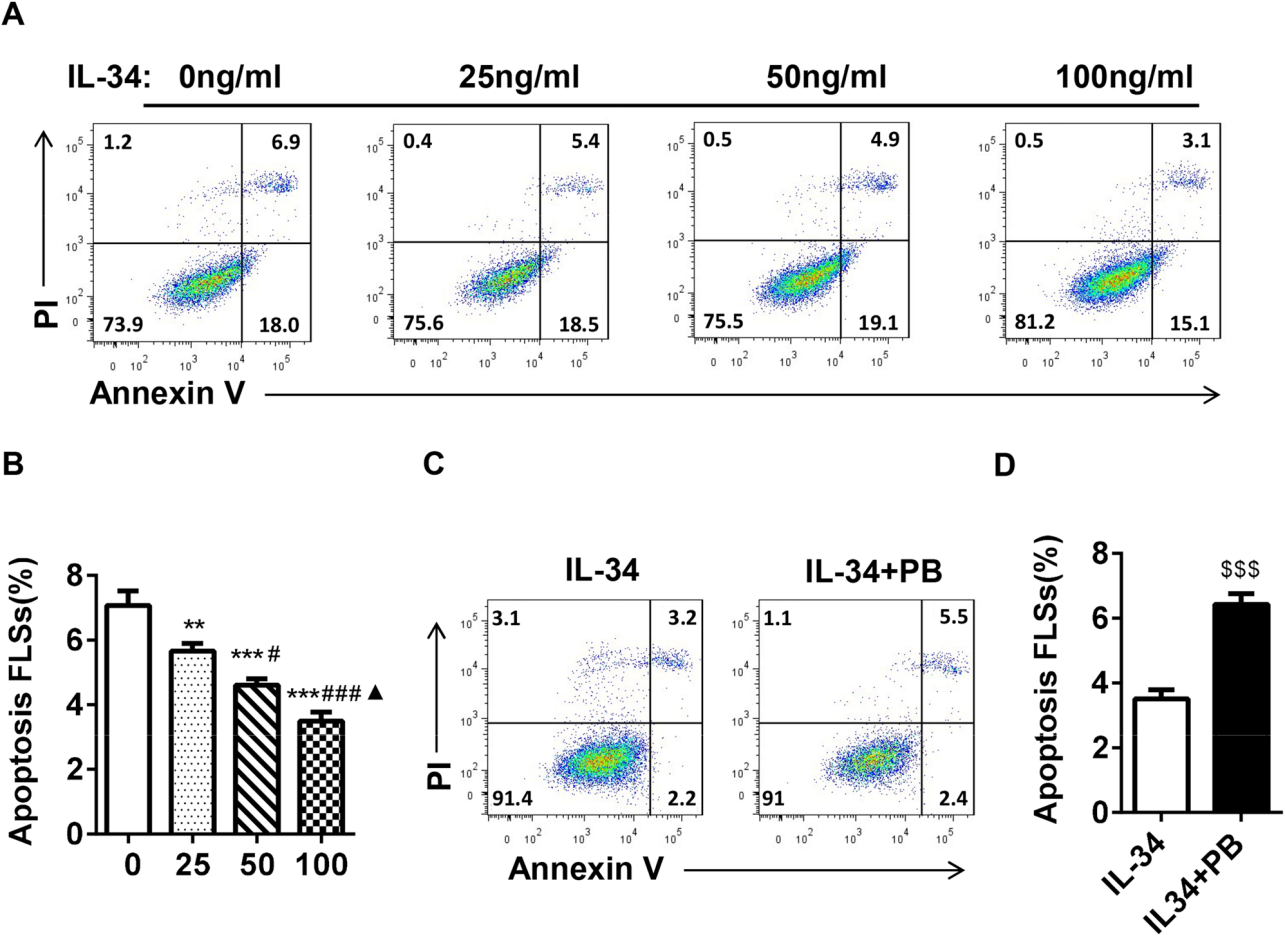
IL-34 inhibited RA-FLS apoptosis by regulating IL-17. (**A**) Apoptosis was analyzed after the treatment of RA-FLSs with different concentrations of IL-34 (0 ng/ml, 25 ng/ml, 50 ng/ml and 100 ng/ml). The degree of apoptosis was assessed by flow cytometry using Annexin V and propidium iodide (PI) staining. (**B**) Quantification of apoptopic RA-FLSs. (**C,D**) Apoptosis was analyzed after the treatment of RA-FLSs with IL-34 (100 ng/ml) or IL-34 (100 ng/ml) + PB (4 UM). The differences between the four groups were tested by ANOVA. Statistical comparisons between two groups were performed by t-tests. The data are expressed as the mean ± SEM. **P < 0.01, ***P < 0.001 vs the 0 group; ^#^*P* < 0.05, ^###^*P* < 0.001 vs the 25 group; ^▲^*P* < 0.05 vs the 50 group; ^$$$^*P* < 0.001 vs the IL-34 group.

To further investigate whether IL-34 increases cell proliferation by inhibiting apoptosis, RA-FLSs were treated with different concentrations of IL-34 for 48 h and harvested. The cells were stained with Annexin V and PI and analyzed by flow cytometry. There were significant differences in the percentages of apoptotic cells in the absence of IL-34 (7.07% ± 0.4%) and after treatment with 25 ng/ml IL-34 (5.66% ± 0.2%), 50 ng/ml IL-34 (4.61% ± 0.2%) or 100 ng/ml IL-34 (3.50% ± 0.3%) (Fig. 2A,B). Furthermore, we used PB in this assay and found that PB robustly inhibited the effect of IL-34, with a significant difference in the percentage of apoptotic cells (3.52% ± 0.3% vs 6.43% ± 0.3%) (Fig. 2C,D). These data indicated that IL-34 decreased apoptosis, and inhibiting IL-17A reversed this effect.

### IL-34 upregulated the mRNA expression of IL-17A, IL-6, TNF-α, VEGF and HIF-1α by regulating IL-17

**Figure 3:**
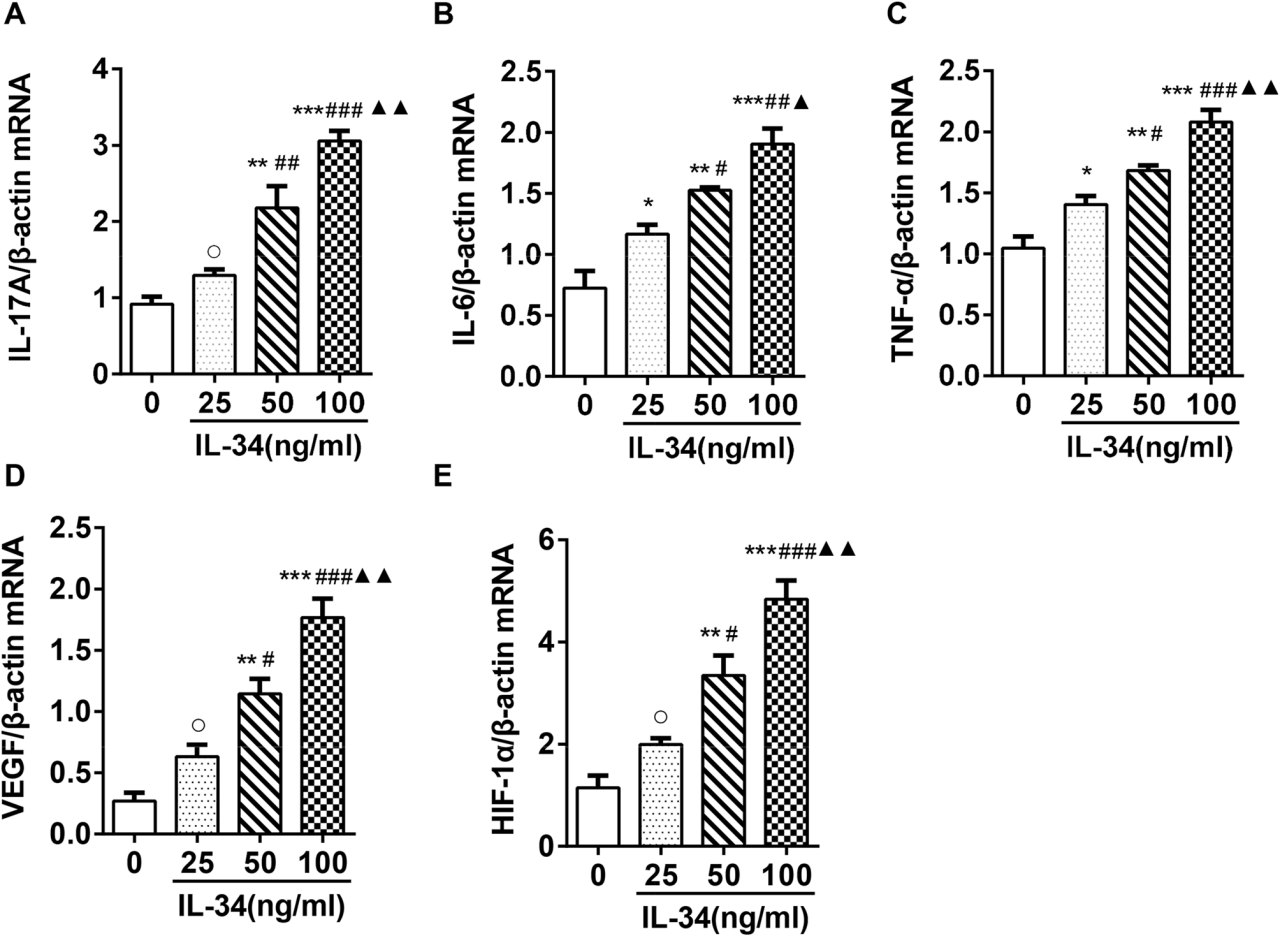
IL-34 upregulated the mRNA expression of IL-17A, IL-6, TNF-α, VEGF and HIF-1α in RA-FLSs. (**A–E**) RT-PCR was used to determine IL-17A,IL-6, TNF-α,VEGF and HIF-1α mRNA levels in RA-FLSs after treatment with IL-34 (0 ng/ml, 25 ng/ml, 50 ng/ml and 100 ng/ml). The differences between the four groups were tested by ANOVA. The data are expressed as the mean ± SEM. *P < 0.05, **P < 0.01, ***P < 0.001 vs the 0 group; ^#^*P* < 0.05, ^##^*P* < 0.01, ^###^*P* < 0.001 vs the 25 group; ^▲^*P* < 0.05, ^▲▲^*P* < 0.01 vs the 50 group; ^○^*P* > 0.05 vs 0 the group.

**Figure 4:**
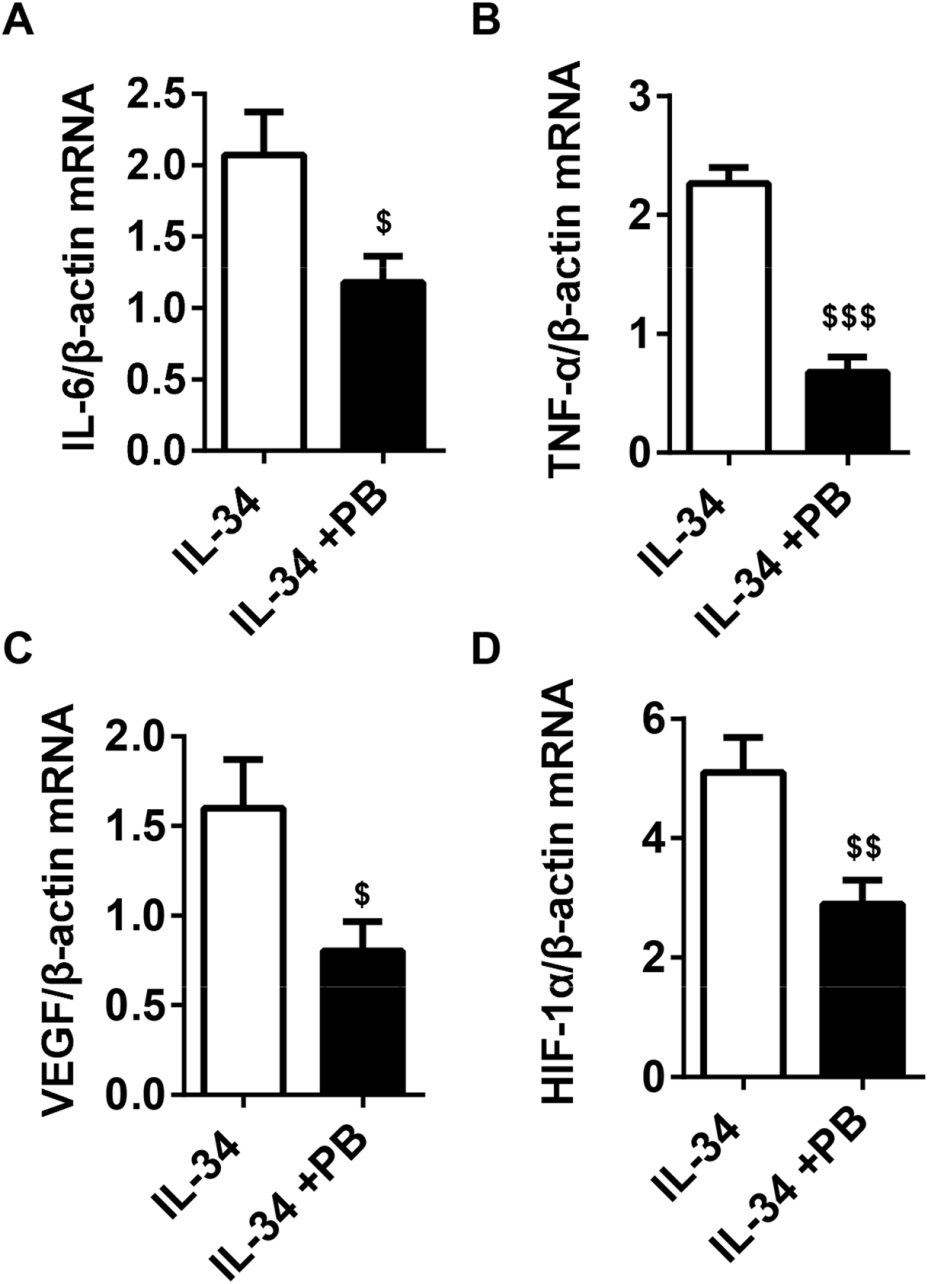
PB antagonized the effect of IL-34 on the mRNA expression of IL-6, TNF-α, VEGF, and HIF-1α in RA-FLSs. (**A–D**) RT-PCR analysis to determine IL-6, TNF-α, VEGF and HIF-1α mRNA levels in RA-FLSs after treatment with IL-34 (100 ng/ml) and IL-34 (100 ng/ml) + PB (4 µM). The differences between two groups were tested by two-tailed Student’s t-tests. The data are expressed as the mean ± SEM. ^$^*P* < 0.05, ^$$^*P* < 0.01, ^$$$^*P* < 0.001 vs the IL-34 group.

We further investigated the effects of IL-34 on cytokine expression in RA-FLSs and showed that IL-34 significantly increased the mRNA expression of IL-17A, IL-6, TNF-α, VEGF and HIF-1α (Fig. 3A–E). To investigate the role of IL-17A in the expression of cytokines in RA-FLSs, we treated IL-34-stimulated RA-FLSs with or without PB. RT-PCR analysis showed that the mRNA expression of IL-6, TNF-α, VEGF and HIF-1α was decreased in RA-FLSs treated withIL-34 + PB (Fig. 4A–D). These results indicate that IL-34 upregulates the mRNA expression of IL-17A, IL-6, TNF-α, VEGF and HIF-1α via IL-17.

### IL-34 upregulated the protein expression of IL-17A, IL-6, TNF-α, VEGF and HIF-1α by regulating IL-17

**Figure 5:**
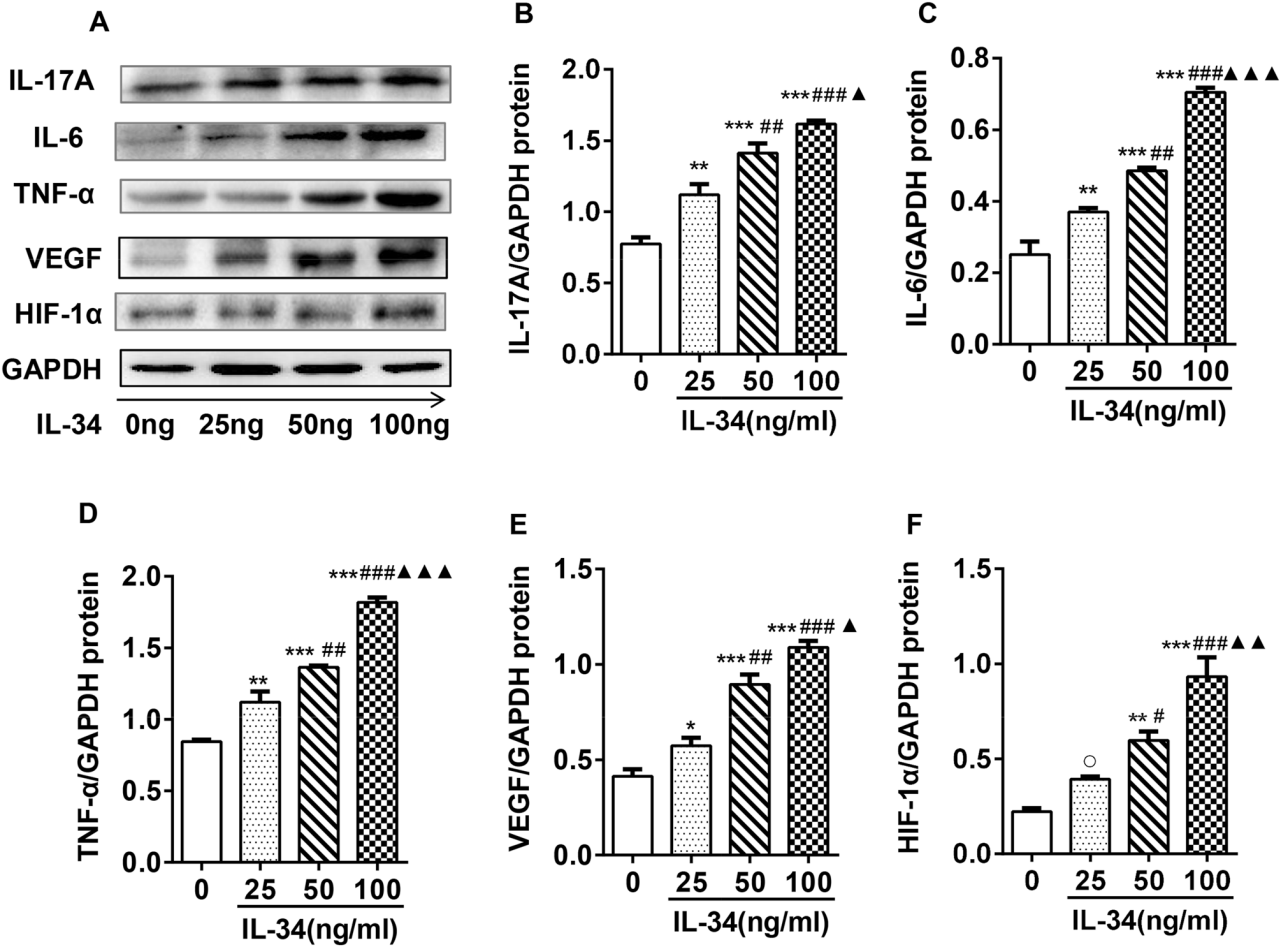
IL-34 upregulated the protein expression of IL-17A, IL-6, TNF-α, VEGF and HIF-1α in RA-FLSs. (**A**) Western blot images of IL-17A, IL-6, TNF-α, VEGF and HIF-1α protein levels in the IL-34 (0 ng/ml, 25 ng/ml, 50 ng/ml and 100 ng/ml) treatment groups. (**B–F**) The protein levels of IL-17A, IL-6, TNF-α, VEGF and HIF-1α in IL-34-treated (0 ng/ml, 25 ng/ml, 50 ng/ml and 100 ng/ml) RA-FLSs were analyzed by Western blotting. The differences between the four groups were tested by ANOVA. The data are expressed as the mean ± SEM. *P < 0.05, **P < 0.01, ***P < 0.001 vs the 0 group; ^#^*P* < 0.05, ^##^*P* < 0.01, ^###^*P* < 0.001 vs the 25 group; ^▲^*P* < 0.05, ^▲▲^*P* < 0.01, ^▲▲▲^*P* < 0.001 vs the 50 group. ^○^*P* > 0.05 vs the 0 group.

**Figure 6:**
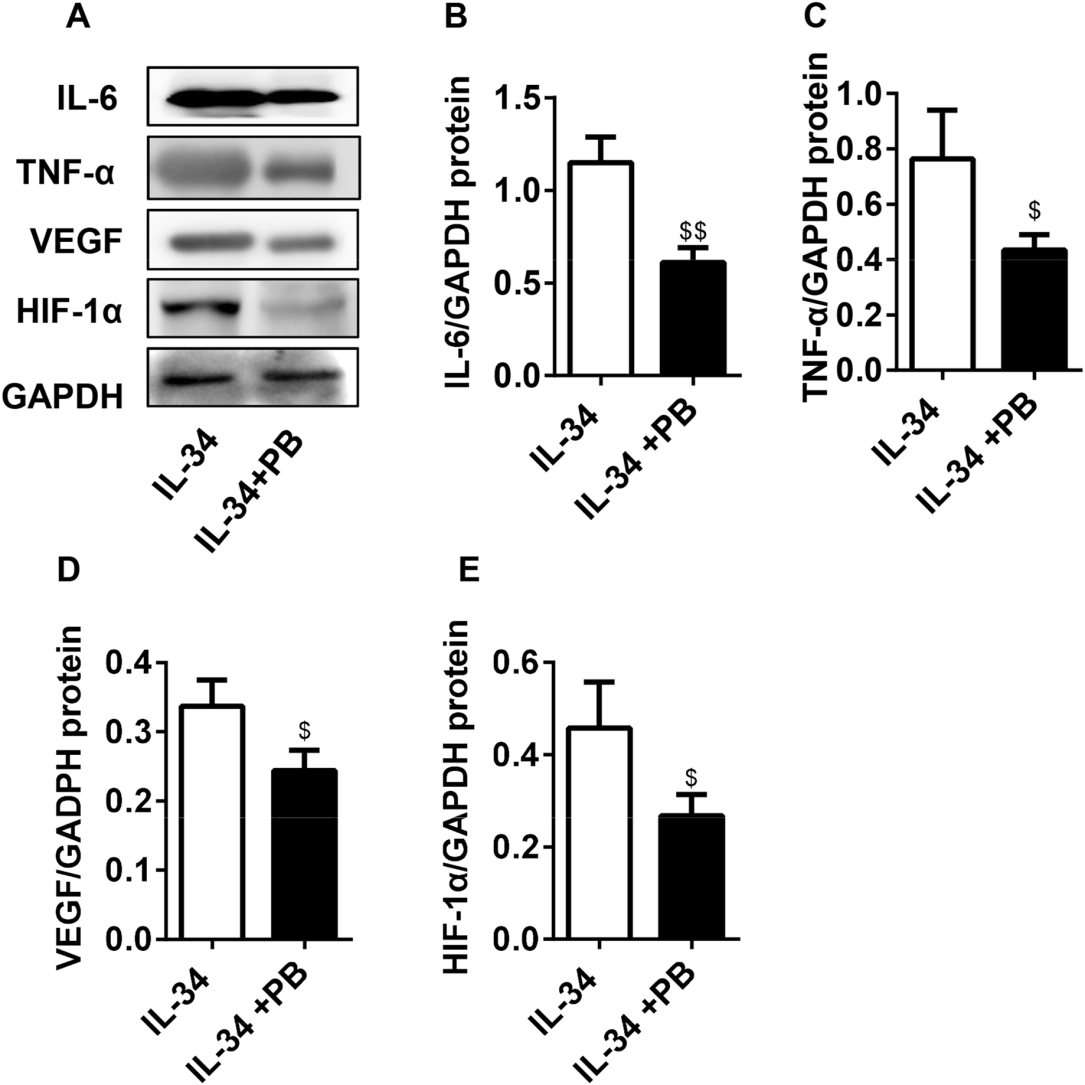
PB antagonized the effect of IL-34 on the protein expression of IL-6, TNF-α, VEGF and HIF-1α in RA-FLSs. (**A**) Western blot images of IL-6,TNF-α,VEGF and HIF-1α protein levels in the IL-34 (100 ng/ml) and IL-34 (100 ng/ml) + PB (4 µM) treatment groups. (**B–E**) The protein levels of IL-6, TNF-α, VEGF and HIF-1α in RA-FLSs treated with IL-34 (100 ng/ml) or IL-34 (100 ng/ml) + PB (4 µM) were analyzed by Western blotting. The differences between two groups were tested by two-tailed Student’s t-tests. The data are expressed as the mean ± SEM. ^$^*P* < 0.05, ^$$^*P* < 0.01 vs the IL-34 group.

Western blot analysis was performed to evaluate the effects of IL-34 on cytokine protein expression in RA-FLSs. As shown in Fig. 5A–F, the protein levels of IL-17A, IL-6, TNF-α, VEGF and HIF-1α were increased in RA-FLSs in response to increasing concentrations of IL-34. Furthermore, Western blot analysis was performed to measure the protein expression in RA-FLSs treated with IL-34 or IL-34 plus PB. We found that inhibiting IL-17A resulted in the inhibition of IL-6, TNF-α, VEGF and HIF-1α protein expression (Fig. 6A–E). Collectively, these findings showed that PB inhibited the protein expression of inflammatory cytokines in IL-34-induced RA-FLSs.

### IL-34 upregulated the secretion of IL-17A, IL-6, TNF-α and VEGF by RA-FLSs by regulating IL-17

**Figure 7:**
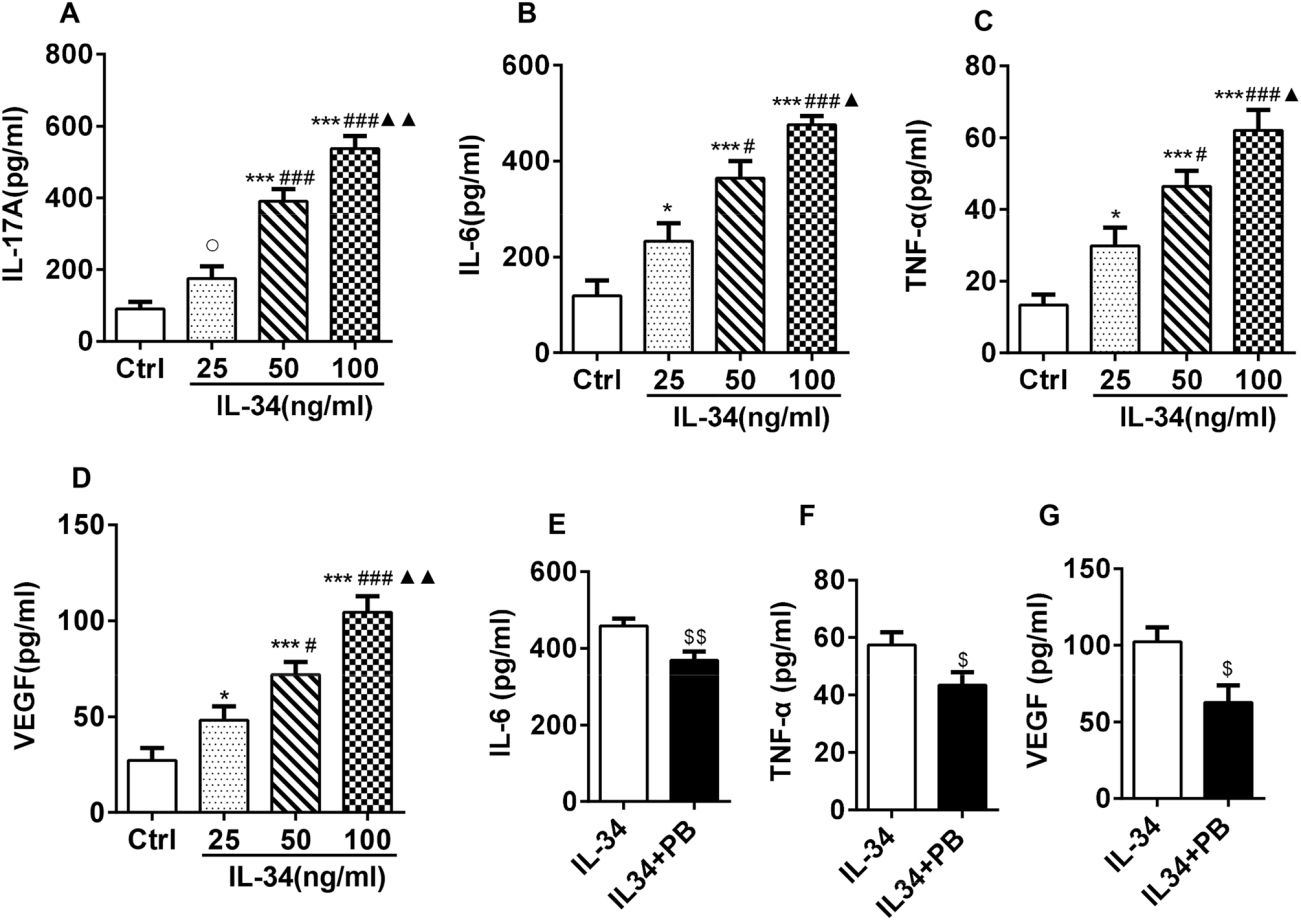
IL-34 upregulated the secretion of IL-17A, IL-6, TNF-α and VEGF in RA-FLSs by regulating IL-17. (**A–D**) RA-FLSs were cultured with different concentrations of IL-34 (0 ng/ml, 25 ng/ml, 50 ng/ml and 100 ng/ml). The levels of IL-17A, IL-6, TNF-α and VEGF in RA-FLS supernatant were determined by ELISA. (**E–G**) RA-FLSs were exposed to 100 ng/ml IL-34 with or without PB. The supernatant was collected for ELISA analysis of IL-6, TNF-α and VEGF levels. The differences between the four groups were tested by ANOVA. The differences between two groups were tested by two-tailed Student’s t-tests. The data are expressed as the mean ± SEM. ^$^*P* < 0.05, ^$$^*P* < 0.01 vs the IL-34 group.

The effects of IL-34 on RA-FLS secretion of proinflammatory cytokines (IL-17, IL-6 and TNF-α) and angiogenic factors (VEGF and HIF-1α) were investigated by ELISA analysis of RA-FLS supernatant. We found that the supernatant levels of IL-17A, IL-6, TNF-α and VEGF were markedly increased with increasing concentrations of IL-34 (Fig. 7A–D). We then found that IL-34-induced secretion of IL-6, TNF-α and VEGF was significantly inhibited by PB (Fig. 7E–G). In addition, the concentration of HIF-1α was lower than the limit of detection of the ELISA kit. Thus, we did not perform any further analyses (data not shown). Taken together, these results suggested that PB also inhibited the secretion of inflammatory cytokines by IL-34-induced RA-FLSs.

## Discussion

RA is a type of chronic systemic inflammatory arthritis characterized by synovitis of the surrounding joints, which can lead to irreversible joint damage and disability. Since continuous joint damage may lead to joint deformity and the loss of joint function^19^, some biomarkers, including cytokines, inflammatory cytokines, chemokines and stromal-degrading enzymes, have been proposed to predict the occurrence and progression of RA^20^. Studies have shown that proinflammatory cytokines such as TNF-α, IL-1, IL-6 and IL-17 play important roles in the pathophysiological development of RA and serve as indicators of therapeutic control for RA patients. These cytokines are involved in and promote osteoclast formation in inflammation and arthritis, leading to the destruction of cartilage and articular bone^21–23^. IL-34 is a proinflammatory factor in RA and is closely associated with the degree of activity in RA^24^. IL-34 produced by human FLSs can promote osteoclast formation^25^. Our research focused on IL-34.

Studies have shown that IL-34 promotes the proliferation and activation of fibroblasts in arthritis^26^. In this study, we showed that IL-34 significantly promoted the proliferation of RA-FLSs and that proliferation increased in a concentration-dependent manner with increasing IL-34 concentrations. Excessive FLS proliferation and insufficient FLS apoptosis are generally considered to be the pathological basis of RA. Our study showed that stimulating RA-FLSs with different concentrations of IL-34 significantly inhibited RA-FLS apoptosis. Moreover, as the IL-34 concentration increased, the apoptosis rate of RA-FLSs decreased.

In people with systemic lupus erythematosus (SLE), IL-34 is expressed by tubular epithelial cells (TECs). IL-34 fosters intrarenal macrophage accumulation via monocyte proliferation in bone marrow and via intrarenal macrophage proliferation. This accumulation leads to macrophage-mediated TEC apoptosis. Expression of IL-34 in TECs correlates with disease activity^27^. Studies have shown that TNF-α induces chemokine production by stimulating synovial and endothelial fibroblast growth factors, and chemokines can activate inflammatory cells to further exacerbate the inflammatory response. In addition, TNF-α can stimulate chondrocytes and synovial fibroblasts to produce collagenase and prostaglandin (PG) E2, inhibit the synthesis of collagen, and promote bone absorption, bone destruction and the proliferation of fibroblasts^28^. In this study, compared with cells in the blank control group, RA-FLSs expressed more TNF-α, IL-6, and IL-17 mRNA and protein with increasing IL-34 concentrations. TNF-α, IL-6 and IL-17 were also secreted into the supernatant by RA-FLSs. Studies have shown that TNF-α can induce the expression of IL-34 through the activation of NF-κB^29^. IL-34 can completely replace M-CSF in the process of RANKL-induced osteoclast formation^30^. This finding indicates that IL-34 and TNF-α have synergistic effects on RA. Our results are consistent with those of Bing Wang and colleagues, who found that the IL-34/CSF-1R axis significantly promotes IL-6 expression in RA-FLSs through the JNK/P38/NF-κB signaling pathway^21^. This finding indicates that IL-34 can promote the occurrence and development of RA by promoting the expression of IL-6.

The relationship between IL-34 and RA angiogenesis has not been clearly reported. However, there is some evidence that IL-34 participates in angiogenesis in RA patients: (1) IL-34 is also expressed on endothelial cells of the lower layer of the synovial lining in RA patients^4^. (2) IL-34 can promote the secretion of VEGF^31^. (3) The M-CSF-IL-34-CSF1R axis is involved in the occurrence, metastasis and angiogenesis of tumors^32,33^. VEGF increases osteoclastogenesis in RA patients and promotes the destruction of joint bone. Juan Zhang reported a strong correlation between the expression of TGF-β and VEGF in RA-FLS supernatant^34^. In the inflammatory state, an increasing number of white blood cells are recruited to the joint, leading to hypoxia, which then leads to the accumulation of HIF-1α in the cytoplasm. HIF-1α is transferred to the nucleus, where HIF-1α and HIF-β and other synergistic stimulators induce macrophages and RA-FLSs to secrete VEGF^16,35^. Under hypoxic conditions, the HIF-1α and VEGF pathways can stimulate angiogenesis through positive feedback regulation^14^. Our previous work showed that the levels of VEGF and HIF-1α were significantly increased in a dose-dependent manner when RA-PBMCs were stimulated with different concentrations of IL-34^19^. In this study, the effects of IL-34 on VEGF and HIF-1α were studied by stimulating RA-FLSs with different concentrations of IL-34. The experimental results showed that compared with those of the blank control group, the mRNA levels and protein levels of VEGF and HIF-1α in RA-FLSs increased with increasing IL-34 concentrations. The level of VEGF secreted by RA-FLS increased significantly in response to difference concentrations of IL-34. These results suggest that IL-34 indirectly causes angiogenesis, promotes the generation of VEGF and HIF-1α, and participates in the pathogenesis of RA.

IL-17 is an inflammatory cytokine secreted primarily by Th17 cells^36^. Th17 cells differentiate from naïve T cells under the influence of Transforming growth factor-beta 1(TGF-β1) and proinflammatory cytokines (IL-1β, IL-6, IL-23)^37^. IL-23 not only stimulates the production of cytokines by differentiated human Th17 cells but also promotes Th17 cell survival^38^. IL-17 was shown to potently induce cytokines such as TNF-a, IL-1β, and granulocyte–macrophage colony-stimulating factor (GM-CSF) from different joint cells as FLS, chondrocytes, and macrophages and was associated to bone and cartilage degradation. The ablation of IL-17 was associated with reduced systemic levels of IL-6 as well as IL-1β and RANKL producing cells in the synovium. Moreover, IL-17A induced neutrophils to produce angiogenic factors^39^. Our previous study showed that in the presence of IL-34, peripheral blood mononuclear cells (PBMCs) from RA patients exhibited increased production of IL-17^2^. These results suggest that IL-34 may play a role through modulating the expression of IL-17. We hypothesized that blocking IL-17 could alter the effect of IL-34 on RA-FLSs. We used an IL-17 inhibitor (PB) to block the effect of IL-17 and observed the effect of IL-34 on RA-FLSs. These results showed that compared with that of RA-FLSs stimulated by IL-34 alone, the proliferation of RA-FLSs stimulated with IL-34 and PB was significantly inhibited, the rate of apoptosis was significantly increased, and the mRNA levels and protein levels of proinflammatory cytokines (IL-6 and TNF-α) and angiogenic factors (VEGF and HIF-1α) decreased significantly. In addition, the RA-FLS secretion of proinflammatory cytokines (IL-6 and TNF-α) and angiogenic factors (VEGF) in the supernatant decreased significantly. These results suggested that IL-34 influenced FLS proliferation, differentiation, apoptosis and secretion of inflammatory cytokines and angiogenic factors by regulating IL-17.

In summary, this study explored the effects of IL-34 on human RA-FLS proliferation, apoptosis, and secretion of inflammatory cytokines and angiogenic factors. An IL-17 inhibitor reversed the effects of IL-34 on FLSs. To our knowledge, there have been no similar previous reports. This research contributes to a better understanding of the mechanism of IL-34 in RA pathology (Supplementary Information).

## Supplementary Information

Supplementary Information.

## Data Availability

The data performed to support the findings of this study are available from the corresponding author upon request.

## References

[1] Ptacek J (2021). Diminished cytokine-induced Jak/STAT signaling is associated with rheumatoid arthritis and disease activity. PLoS ONE.

[2] Li X, Lei Y, Gao Z, Zhang B, Xia L, Lu J, Shen H (2020). Effect of IL-34 on T helper 17 cell proliferation and IL-17 secretion by peripheral blood mononuclear cells from rheumatoid arthritis patients. Sci. Rep..

[3] Garcia S (2016). Colony-stimulating factor (CSF) 1 receptor blockade reduces inflammation in human and murine models of rheumatoid arthritis. Arthritis Res. Ther..

[4] Chemel M (2012). Interleukin 34 expression is associated with synovitis severity in rheumatoid arthritis patients. Ann. Rheum. Dis..

[5] Hwang SJ (2012). Interleukin-34 produced by human fibroblast-like synovial cells in rheumatoid arthritis supports osteoclastogenesis. Arthritis Res. Ther..

[6] Chang SH (2015). Baseline serum interleukin-34 levels independently predict radiographic progression in patients with rheumatoid arthritis. Rheumatol. Int..

[7] Tian Y, Shen H, Xia L, Lu J (2013). Elevated serum and synovial fluid levels of interleukin-34 in rheumatoid arthritis: Possible association with disease progression via interleukin-17 production. J. Interferon Cytokine Res..

[8] Moon SJ, Hong YS, Ju JH, Kwok SK, Park SH, Min JK (2013). Increased levels of interleukin 34 in serum and synovial fluid are associated with rheumatoid factor and anticyclic citrullinated peptide antibody titers in patients with rheumatoid arthritis. J. Rheumatol..

[9] Yang S, Jiang S, Wang Y, Tu S, Wang Z, Chen Z (2016). Interleukin 34 upregulation contributes to the increment of MicroRNA 21 expression through STAT3 activation associated with disease activity in rheumatoid arthritis. J. Rheumatol..

[10] Farah Izati A, Wong KK, Che Maraina CH (2020). IL-23/IL-17 axis in the pathogenesis and treatment of systemic lupus erythematosus and rheumatoid arthritis. Malays. J. Pathol..

[11] Kim KW, Kim BM, Won JY, Min HK, Lee SJ, Lee SH, Kim HR (2020). Tocotrienol regulates osteoclastogenesis in rheumatoid arthritis. Korean J. Intern. Med..

[12] Shi C, Zhang H, Wang X, Jin B, Jia Q, Li Y, Yang Y (2020). Cinnamtannin D1 attenuates autoimmune arthritis by regulating the balance of Th17 and treg cells through inhibition of aryl hydrocarbon receptor expression. Pharmacol. Res..

[13] Saleem B (2012). Can flare be predicted in DMARD treated RA patients in remission, and is it important? A cohort study. Ann. Rheum. Dis..

[14] Hu F (2014). Hypoxia and hypoxia-inducible factor-1α provoke toll-like receptor signalling-induced inflammation in rheumatoid arthritis. Ann. Rheum. Dis.

[15] Londoño D, Cadavid D, Drouin EE, Strle K, McHugh G, Aversa JM, Steere AC (2014). Antibodies to endothelial cell growth factor and obliterative microvascular lesions in the synovium of patients with antibiotic-refractory lyme arthritis. Arthritis Rheumatol..

[16] Elshabrawy HA, Chen Z, Volin MV, Ravella S, Virupannavar S, Shahrara S (2015). The pathogenic role of angiogenesis in rheumatoid arthritis. Angiogenesis.

[17] Clavel G, Bessis N, Boissier MC (2003). Recent data on the role for angiogenesis in rheumatoid arthritis. Jt. Bone Spine.

[18] Iravani Saadi M, Babaee Beigi MA, Ghavipishe M, Tahamtan M, Geramizadeh B, Zare A, Yaghoobi R (2019). The circulating level of interleukins 6 and 18 in ischemic and idiopathic dilated cardiomyopathy. J. Cardiovasc. Thorac Res..

[19] Ding LL, Li X, Lei YM, Xia LP, Lu J, Shen H (2020). Effect of interleukin-34 on secretion of angiogenesis cytokines by peripheral blood mononuclear cells of rheumatoid arthritis. Immunol. Investig..

[20] MacDonald IJ, Huang CC, Liu SC, Tang CH (2020). Reconsidering the role of melatonin in rheumatoid arthritis. Int. J. Mol. Sci..

[21] Wang B (2017). IL-34 Upregulated Th17 Production through increased IL-6 expression by rheumatoid fibroblast-like synoviocytes. Mediat. Inflamm..

[22] Chemel M (2017). Bone morphogenetic protein 2 and transforming growth factor β1 inhibit the expression of the proinflammatory cytokine IL-34 in rheumatoid arthritis synovial fibroblasts. Am. J. Pathol..

[23] Xing R (2016). Interleukin-21 induces proliferation and proinflammatory cytokine profile of fibroblast-like synoviocytes of patients with rheumatoid arthritis. Scand. J. Immunol..

[24] Zhou RP, Wu XS, Xie YY, Dai BB, Hu W, Ge JF, Chen FH (2016). Functions of interleukin-34 and its emerging association with rheumatoid arthritis. Immunology.

[25] Baghdadi M (2019). A role for IL-34 in osteolytic disease of multiple myeloma. Blood Adv..

[26] Galligan CL, Fish EN (2017). Interleukin-34 promotes fibrocyte proliferation. J. Interferon Cytokine Res..

[27] Wada Y, Gonzalez-Sanchez HM, Weinmann-Menke J, Iwata Y, Ajay AK, Meineck M, Kelley VR (2019). IL-34-Dependent intrarenal and systemic mechanisms promote lupus nephritis in MRL-Fas(lpr) mice. J. Am. Soc. Nephrol..

[28] Karaahmet OZ, Bal A, Dulgeroglu D, Gurcay E, Gezer HH, Cakci A (2016). Effect of exposure to tobacco smoke on response to anti-tumor necrosis factor-alpha treatment in patients with rheumatoid arthritis. Iran. J. Public Health.

[29] Yu Y, Yang D, Qiu L, Okamura H, Guo J, Haneji T (2014). Tumor necrosis factor-α induces interleukin-34 expression through nuclear factor-κB activation in MC3T3-E1 osteoblastic cells. Mol. Med. Rep..

[30] Boström EA, Lundberg P (2013). The newly discovered cytokine IL-34 is expressed in gingival fibroblasts, shows enhanced expression by pro-inflammatory cytokines, and stimulates osteoclast differentiation. PLoS ONE.

[31] Eda H, Zhang J, Keith RH, Michener M, Beidler DR, Monahan JB (2010). Macrophage-colony stimulating factor and interleukin-34 induce chemokines in human whole blood. Cytokine.

[32] Ségaliny AI (2015). Interleukin-34 promotes tumor progression and metastatic process in osteosarcoma through induction of angiogenesis and macrophage recruitment. Int. J. Cancer.

[33] Wang B, Xu W, Tan M, Xiao Y, Yang H, Xia TS (2015). Integrative genomic analyses of a novel cytokine, interleukin-34 and its potential role in cancer prediction. Int. J. Mol. Med..

[34] Zhang J, Li C, Zheng Y, Lin Z, Zhang Y, Zhang Z (2017). Inhibition of angiogenesis by arsenic trioxide via TSP-1-TGF-β1-CTGF-VEGF functional module in rheumatoid arthritis. Oncotarget.

[35] Konisti S, Kiriakidis S, Paleolog EM (2012). Hypoxia—A key regulator of angiogenesis and inflammation in rheumatoid arthritis. Nat. Rev. Rheumatol..

[36] Schminke B, Trautmann S, Mai B, Miosge N, Blaschke S (2016). Interleukin 17 inhibits progenitor cells in rheumatoid arthritis cartilage. Eur. J. Immunol..

[37] Volpe E, Servant N, Zollinger R, Bogiatzi SI, Hupé P, Barillot E, Soumelis V (2008). A critical function for transforming growth factor-beta, interleukin 23 and proinflammatory cytokines in driving and modulating human T(H)-17 responses. Nat. Immunol..

[38] Blauvelt A, Chiricozzi A (2018). The immunologic role of IL-17 in psoriasis and psoriatic arthritis pathogenesis. Clin. Rev. Allergy Immunol..

[39] Zwicky P, Unger S, Becher B (2020). Targeting interleukin-17 in chronic inflammatory disease: A clinical perspective. J. Exp. Med..

