# Supplementary material for: IL-34 affects fibroblast-like synoviocyte proliferation, apoptosis and function by regulating IL-17
Source: Sci Rep. 2021 Aug 12;11:16378. doi: 10.1038/s41598-021-95839-1 (PMC8361173; doi:10.1038/s41598-021-95839-1)
Supplement: Supplementary file 1 — Supplementary Information. [file 41598_2021_95839_MOESM1_ESM.docx]

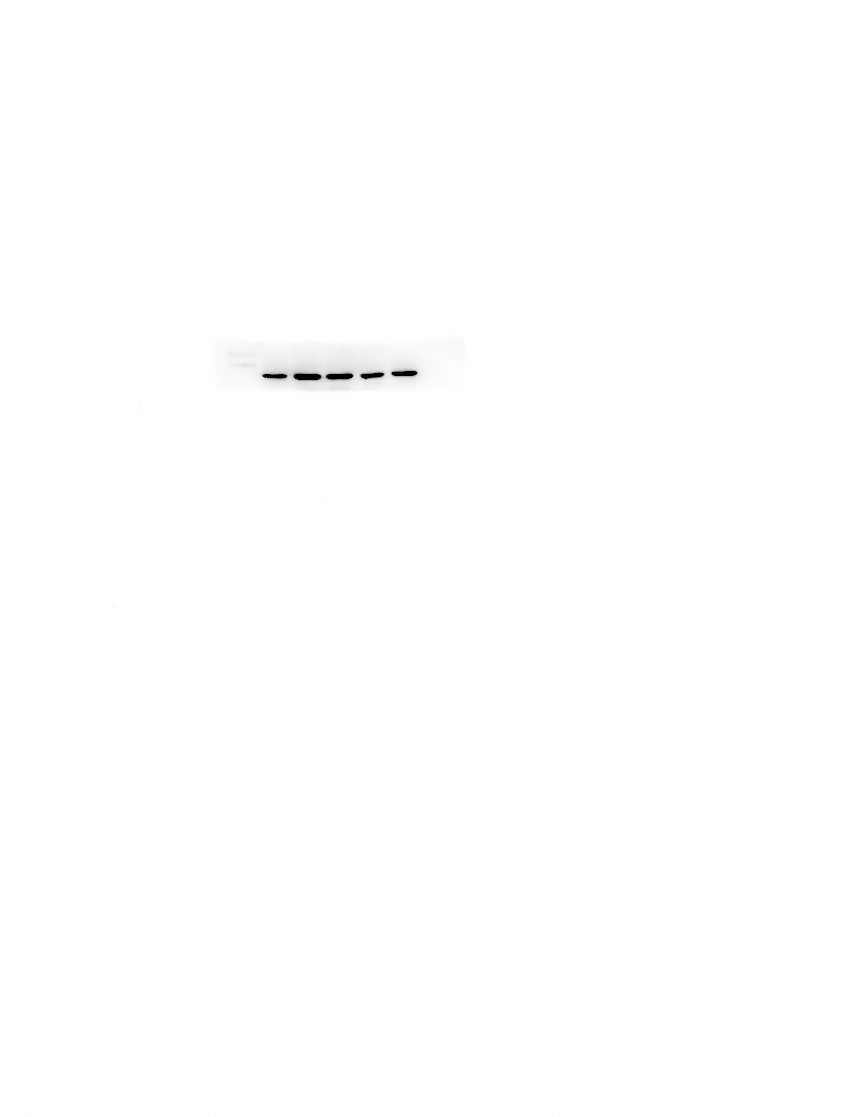


IL-34(ng/ml): 0, 25 50 ,100 100+PB


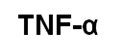


IL-34(ng/ml): 0 25 50 100 100+PB


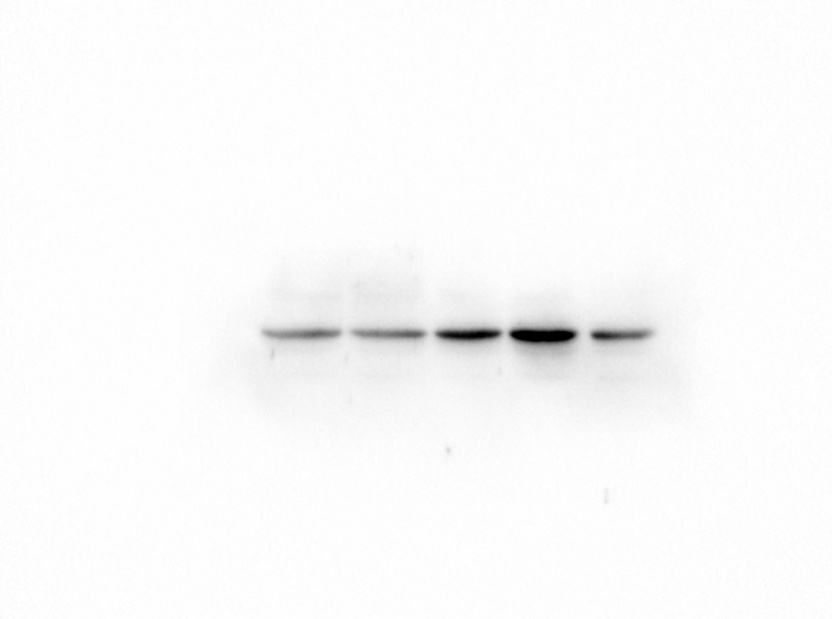


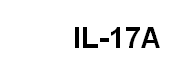


IL-34(ng/ml): 0 25 50 100 100+PB


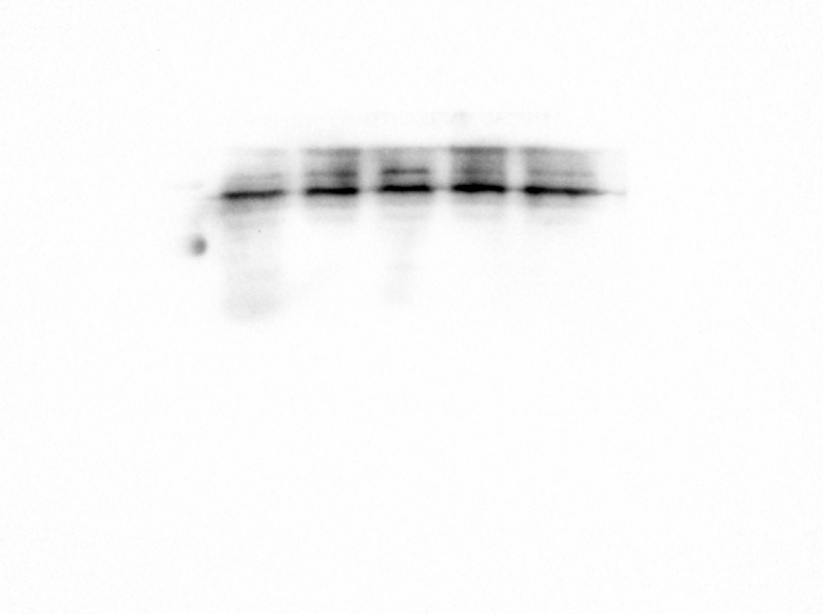


**HIF-1a**

IL-34(ng/ml):Marker 0 25 50 100 100+PB

50 100 100+PB


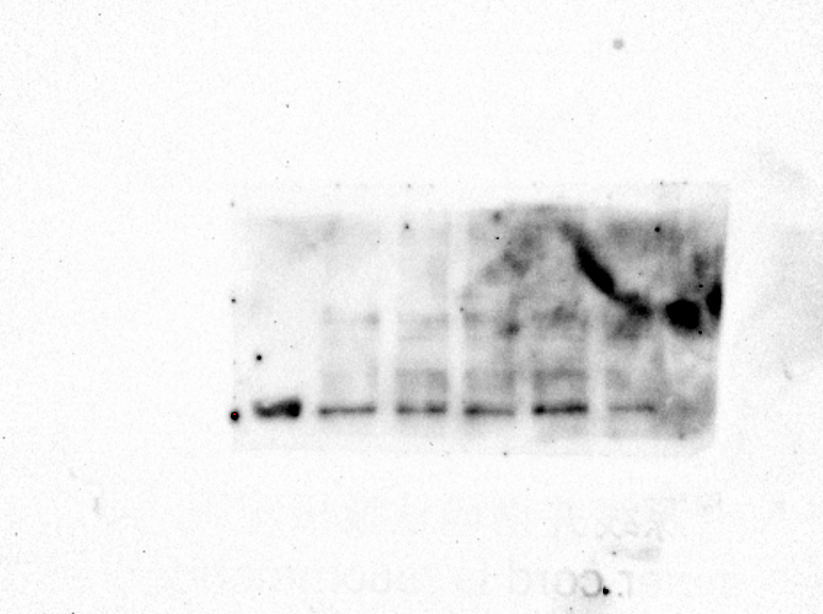


**VEGF**

IL-34(ng/ml): 0 25 50 100 100+PB


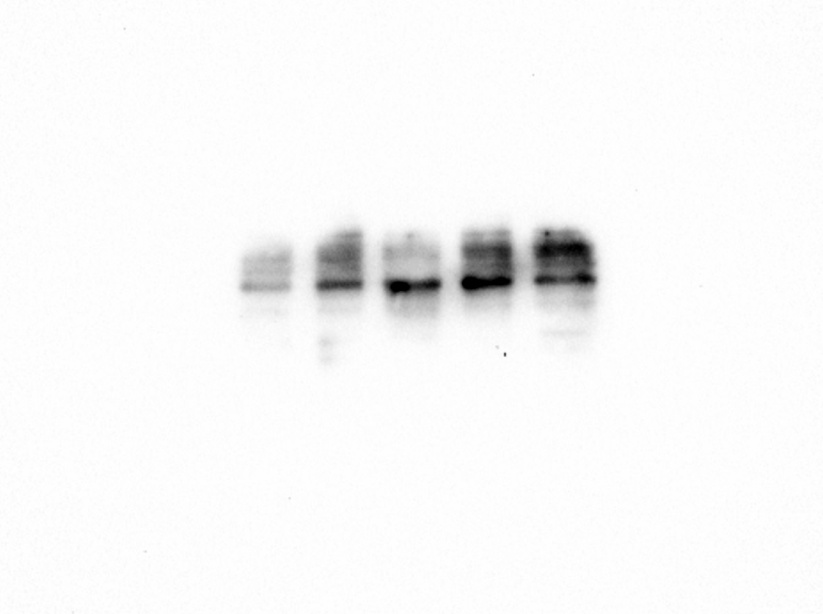


**IL-6**

IL-34(ng/ml): 0 25 50 100


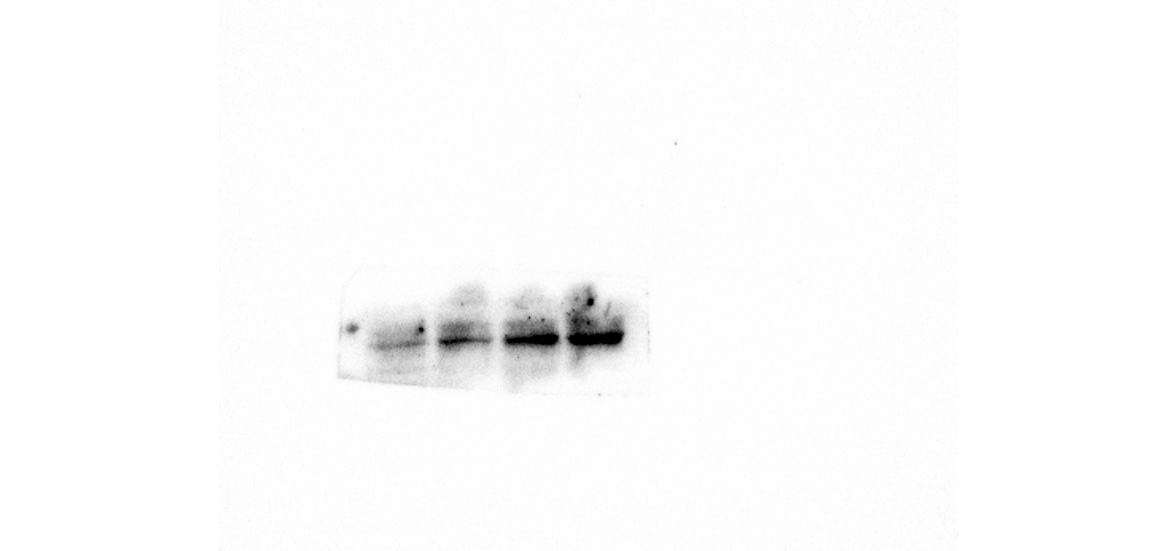


**TNF-a**

IL-34(ng/ml): 0 25 50 100 100+PB


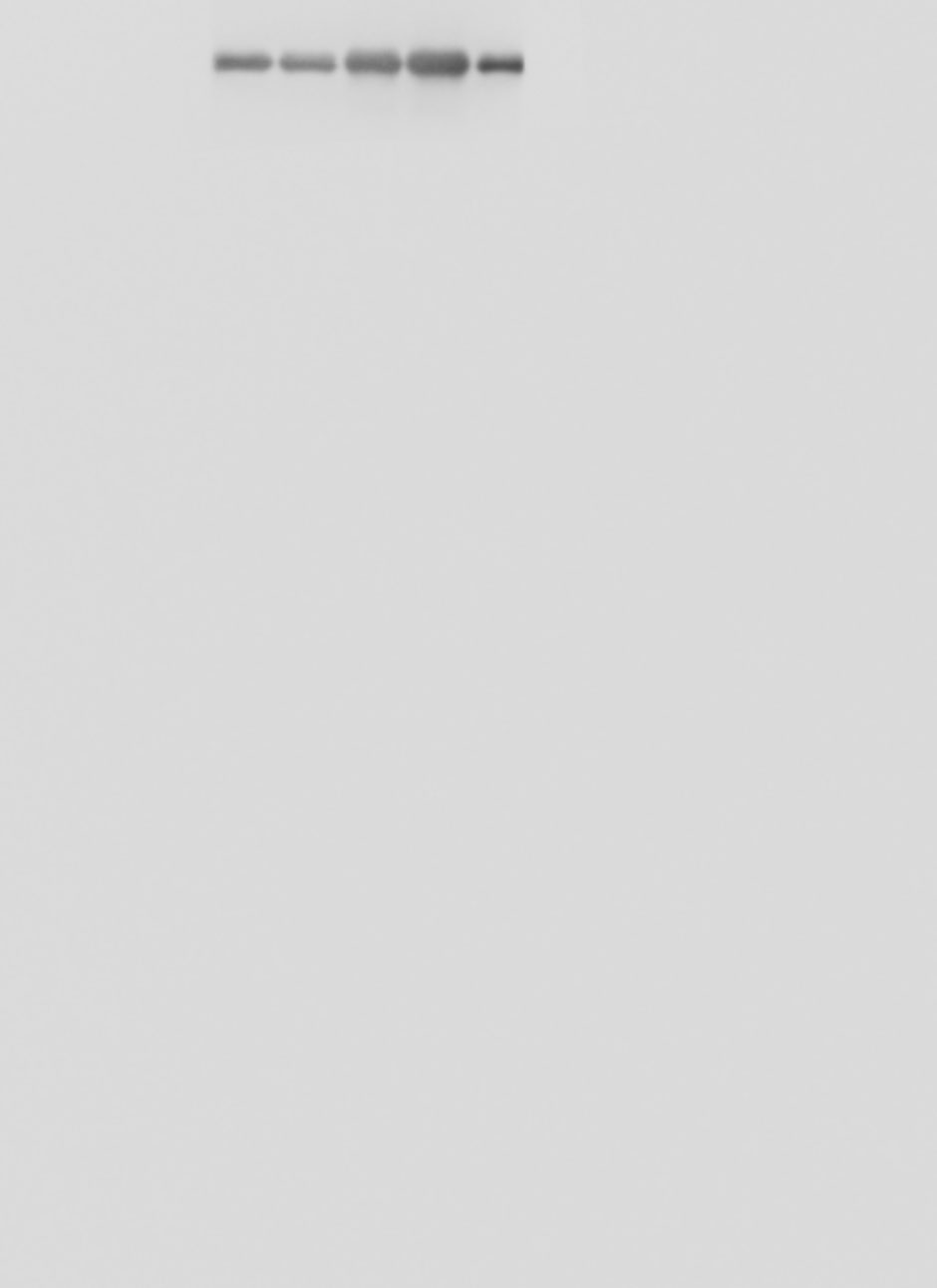


**GAPDH**

IL-34(ng/ml): 0 25 50 100 100+PB


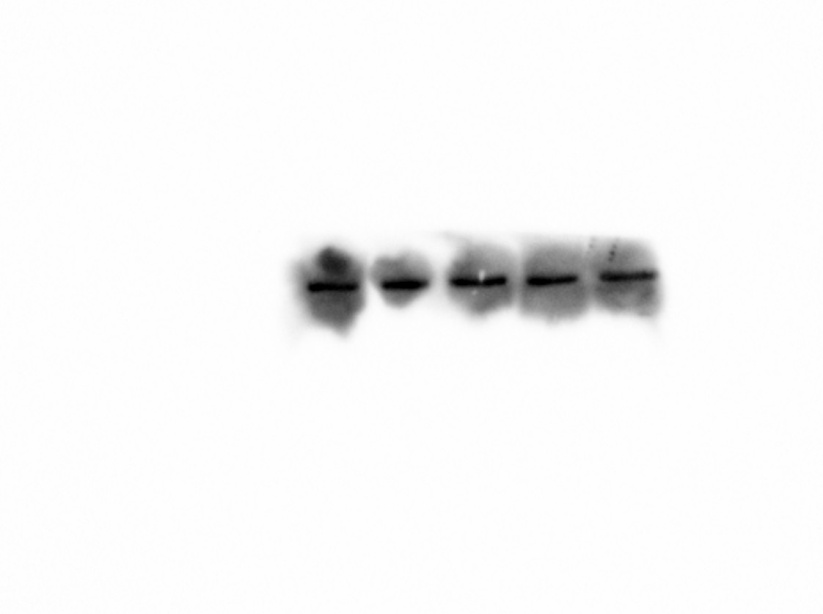


**IL-6**

IL-34(ng/ml): 100 100+PB


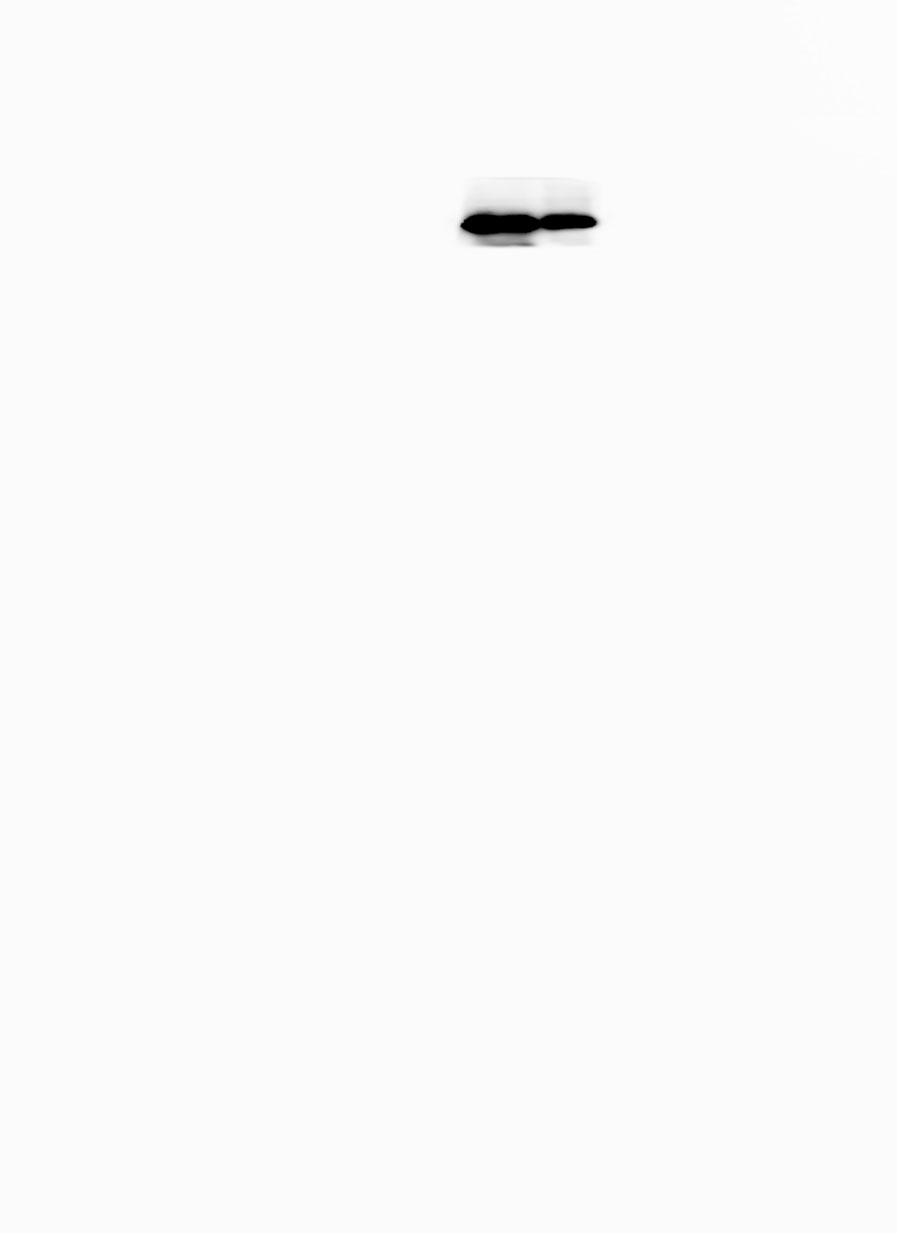


**VEGF**

IL-34(ng/ml): 100 100+PB


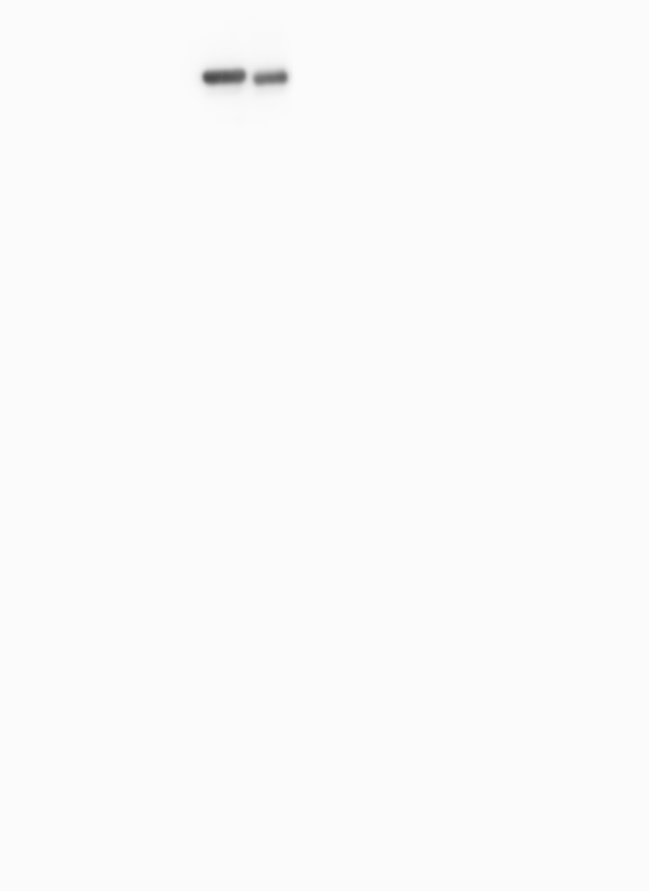


HIF-1α

IL-34(ng/ml): 100 100+PB


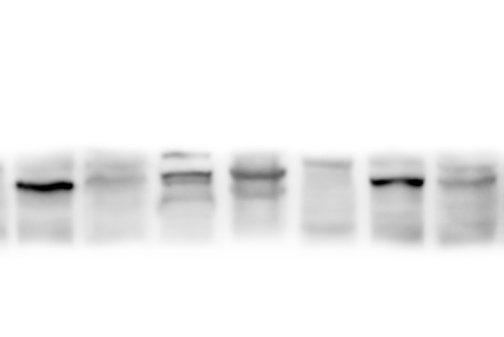


**GAPDH**

IL-34(ng/ml): 0 25 50 100


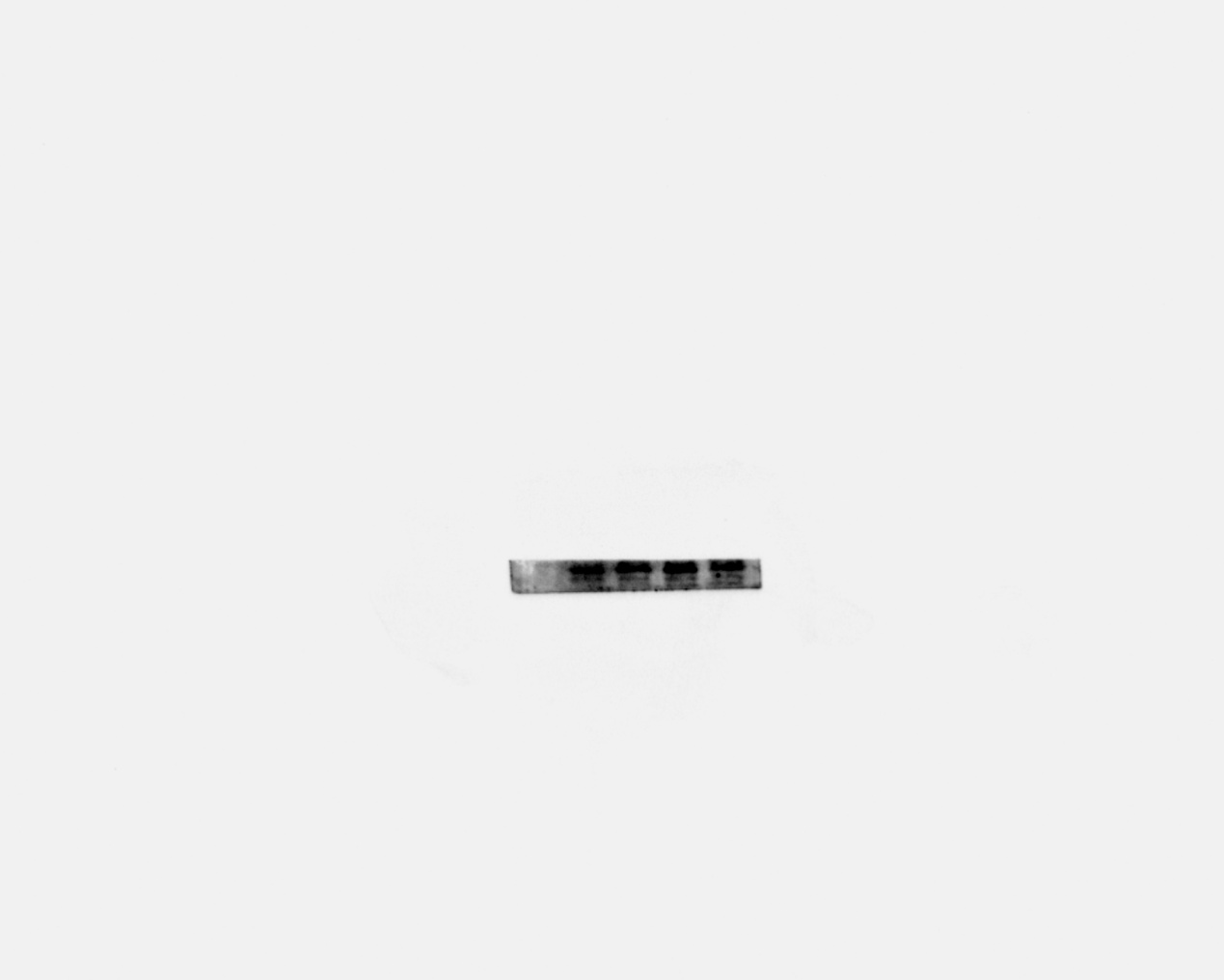


**GAPDH**

IL-34(ng/ml): 0 25 50 100


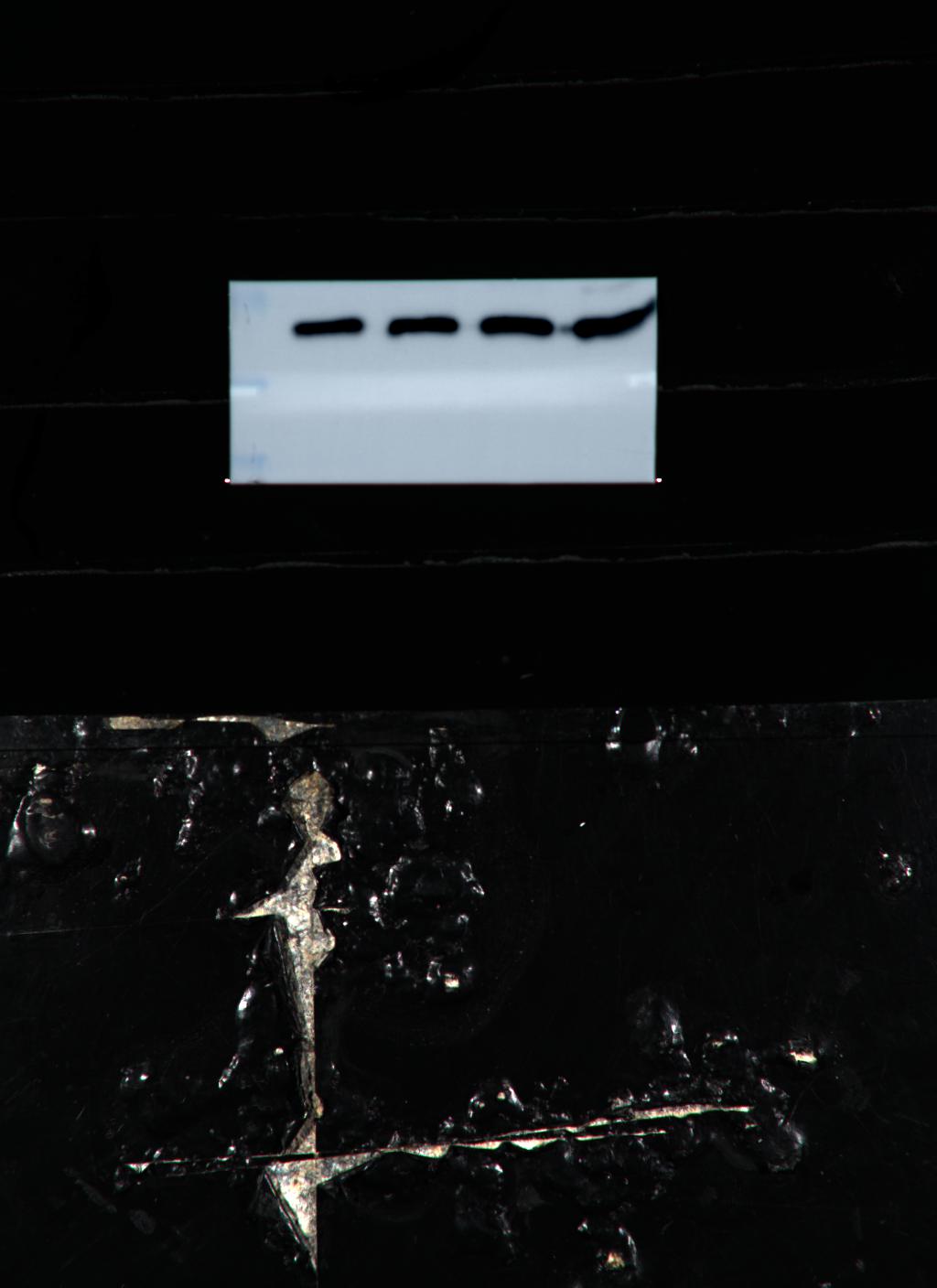


**GAPDH**

IL-34(ng/ml): 0 25 50 100


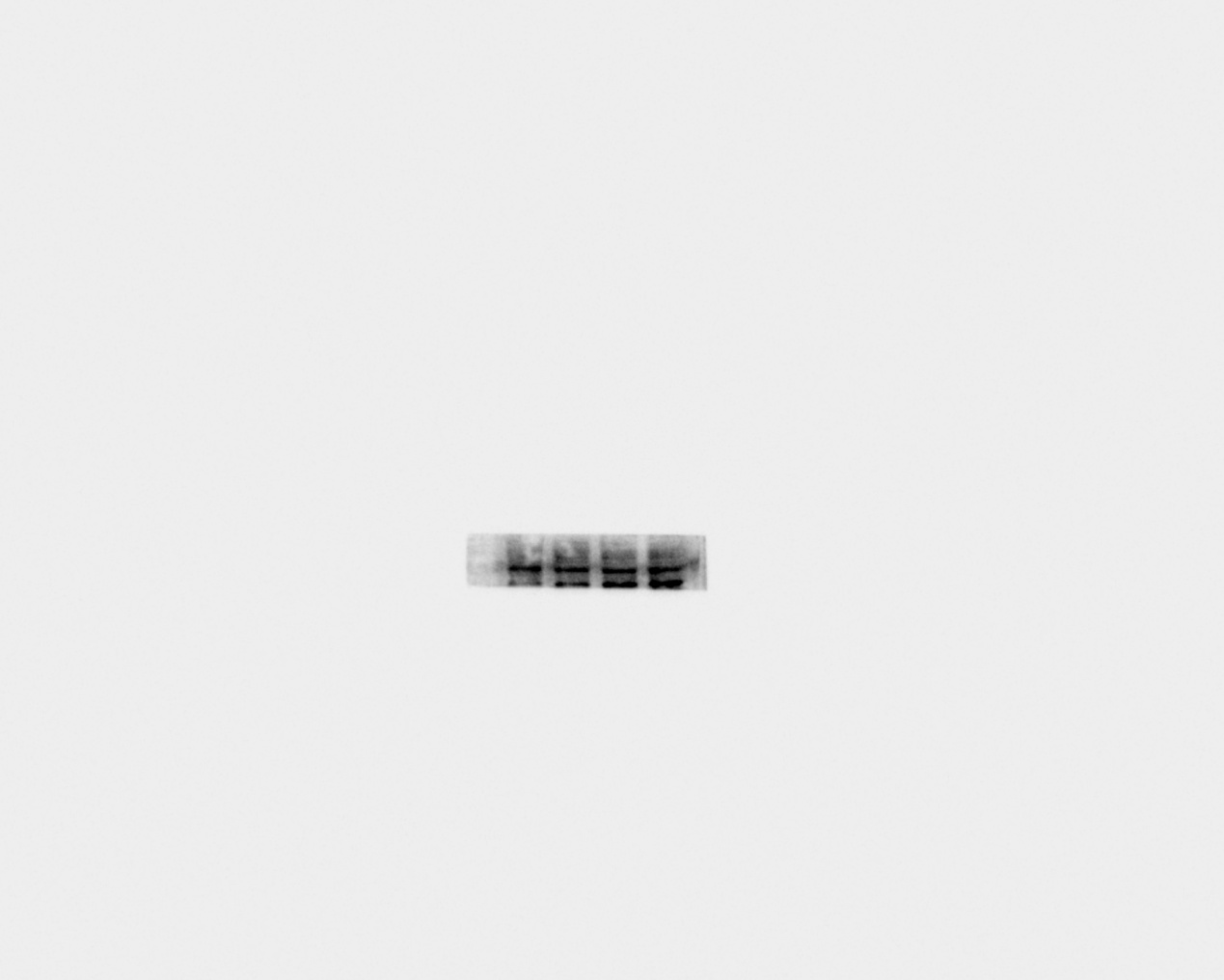


**TNF-α**

IL-34(ng/ml): 0 25 50 100


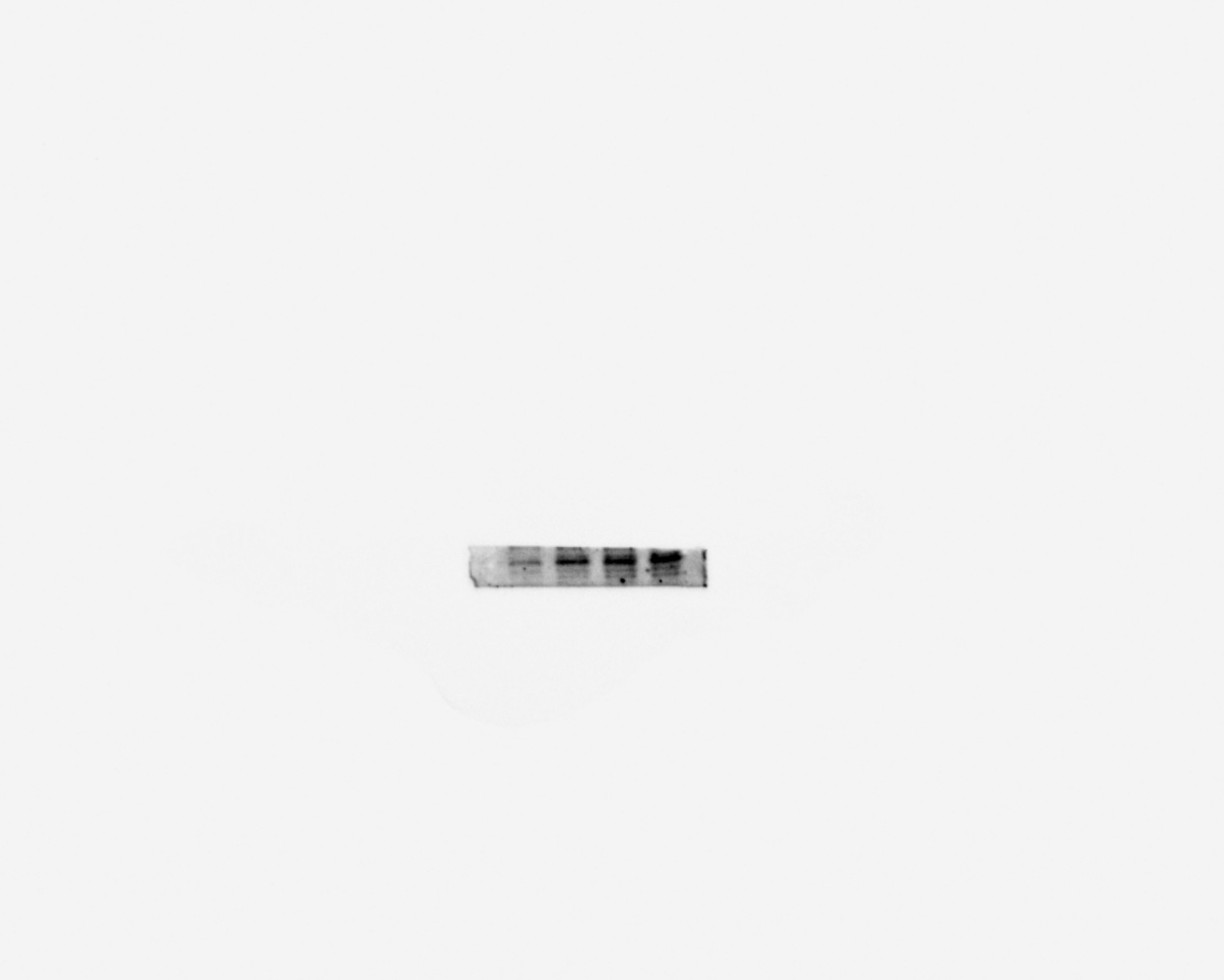


**TNF-α**

IL-34(ng/ml): 0 25 50 100


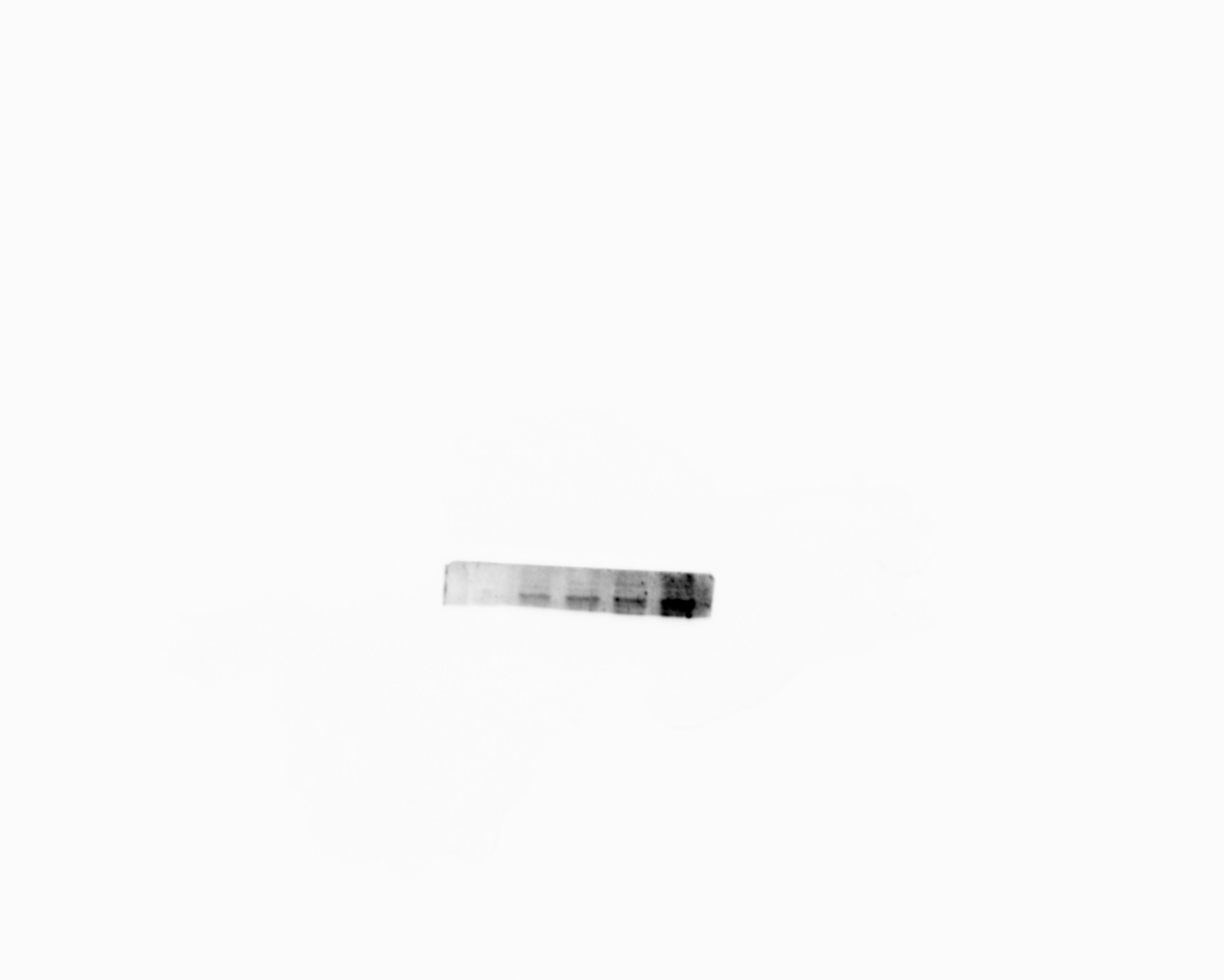


**IL-17A**

IL-34(ng/ml): 0 25 50 100


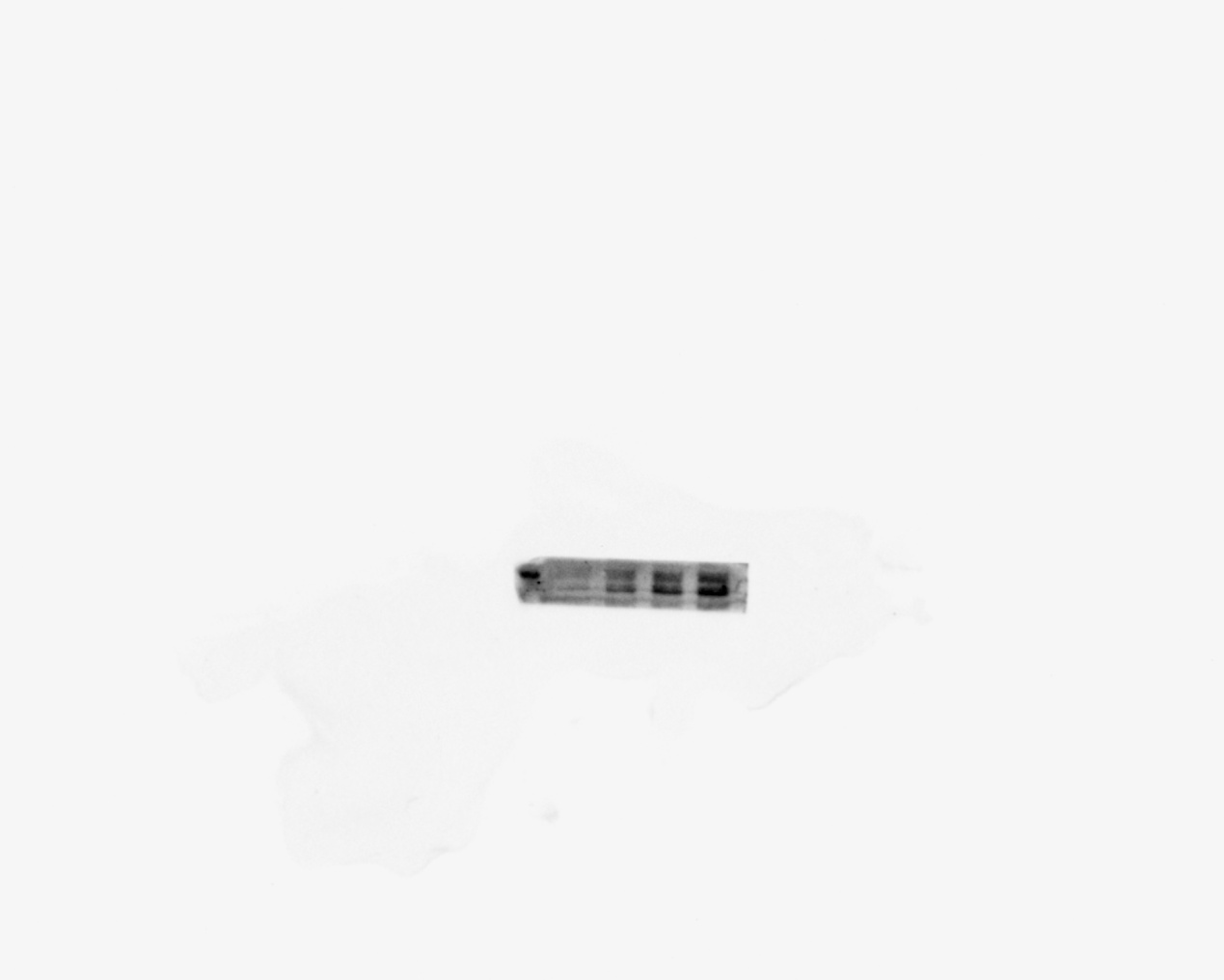


**IL-17A**

IL-34(ng/ml): 0 25 50 100


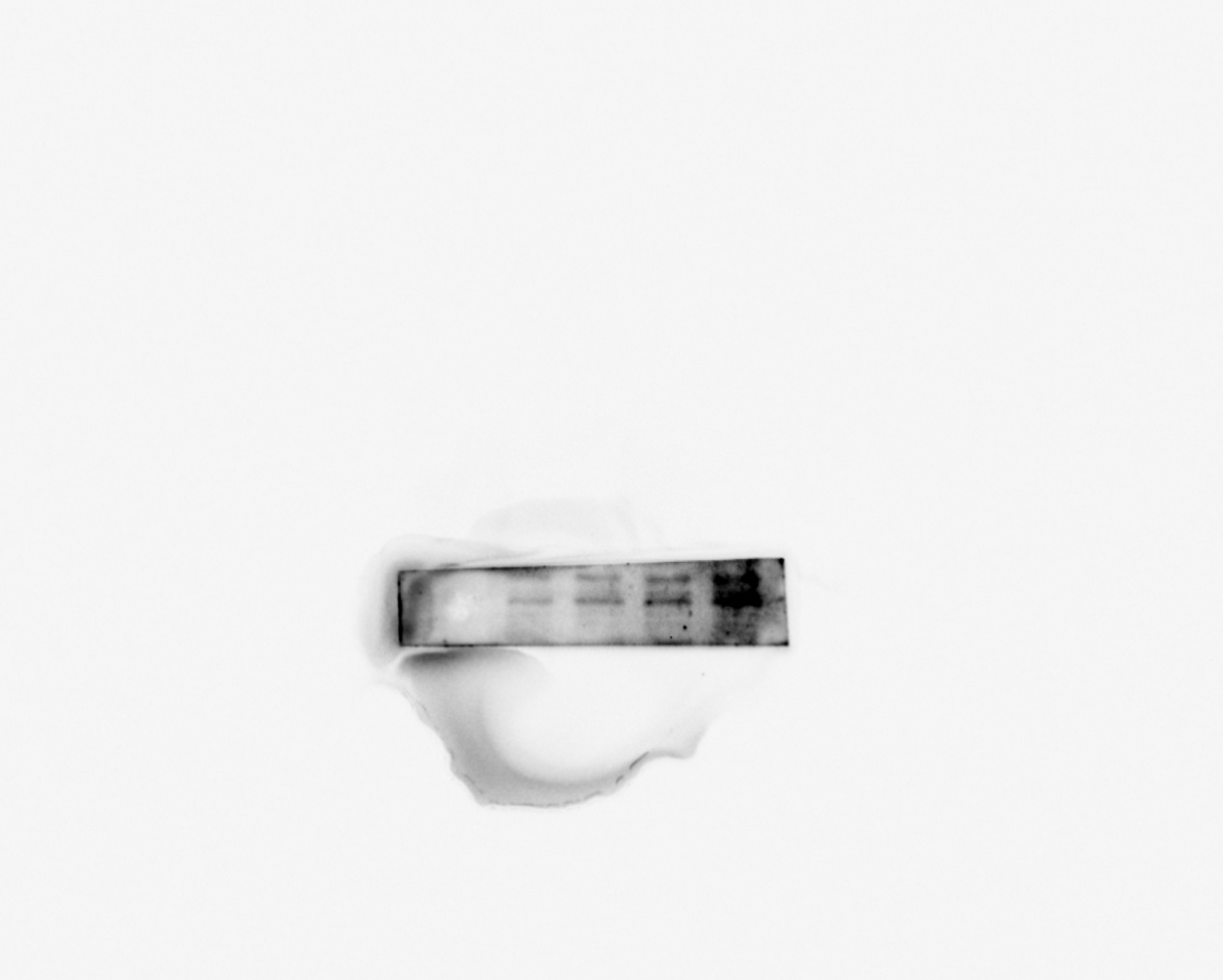


**HIF-1a**

IL-34(ng/ml): 0 25 50 100


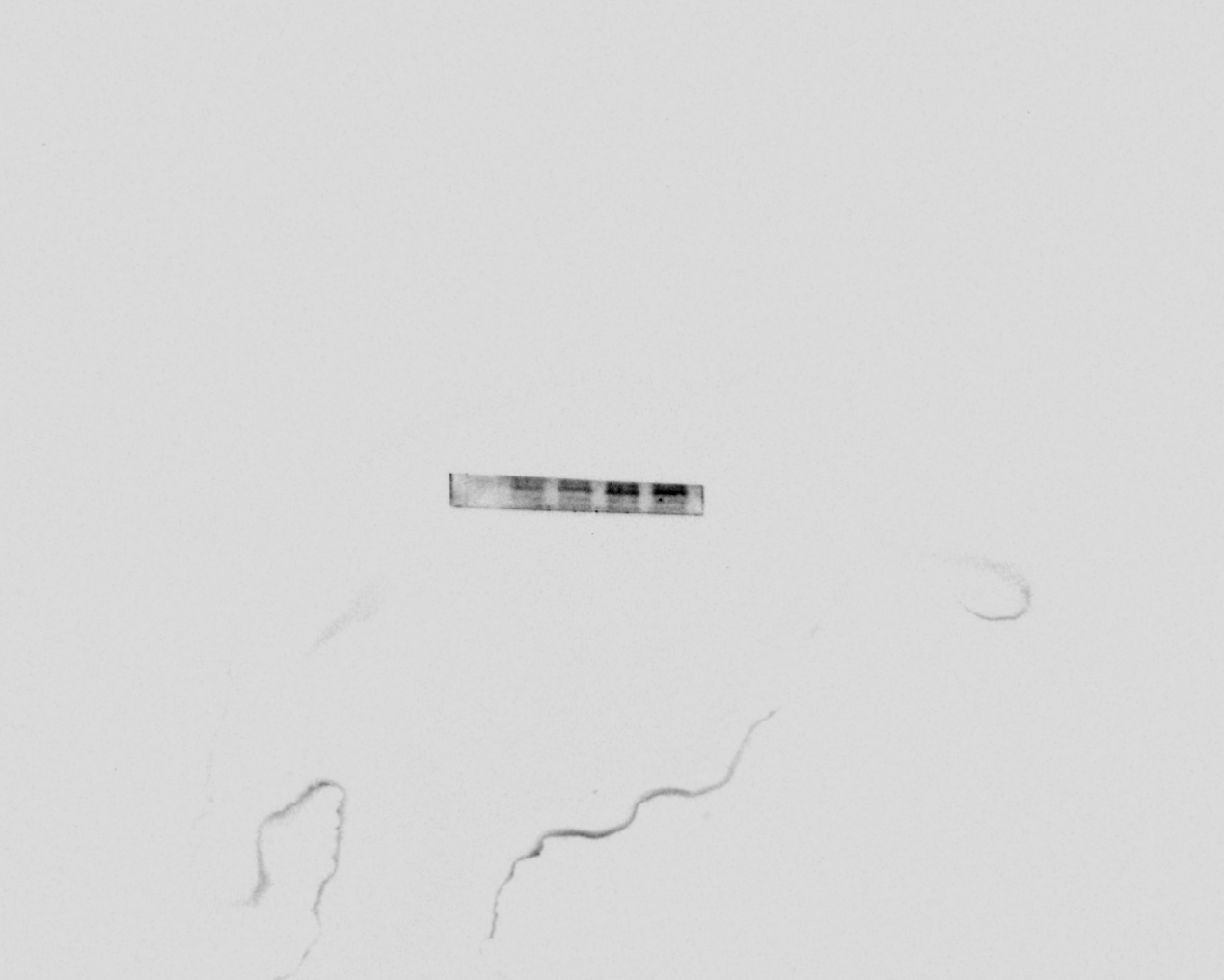


**HIF-1a**

IL-34(ng/ml): 0 25 50 100


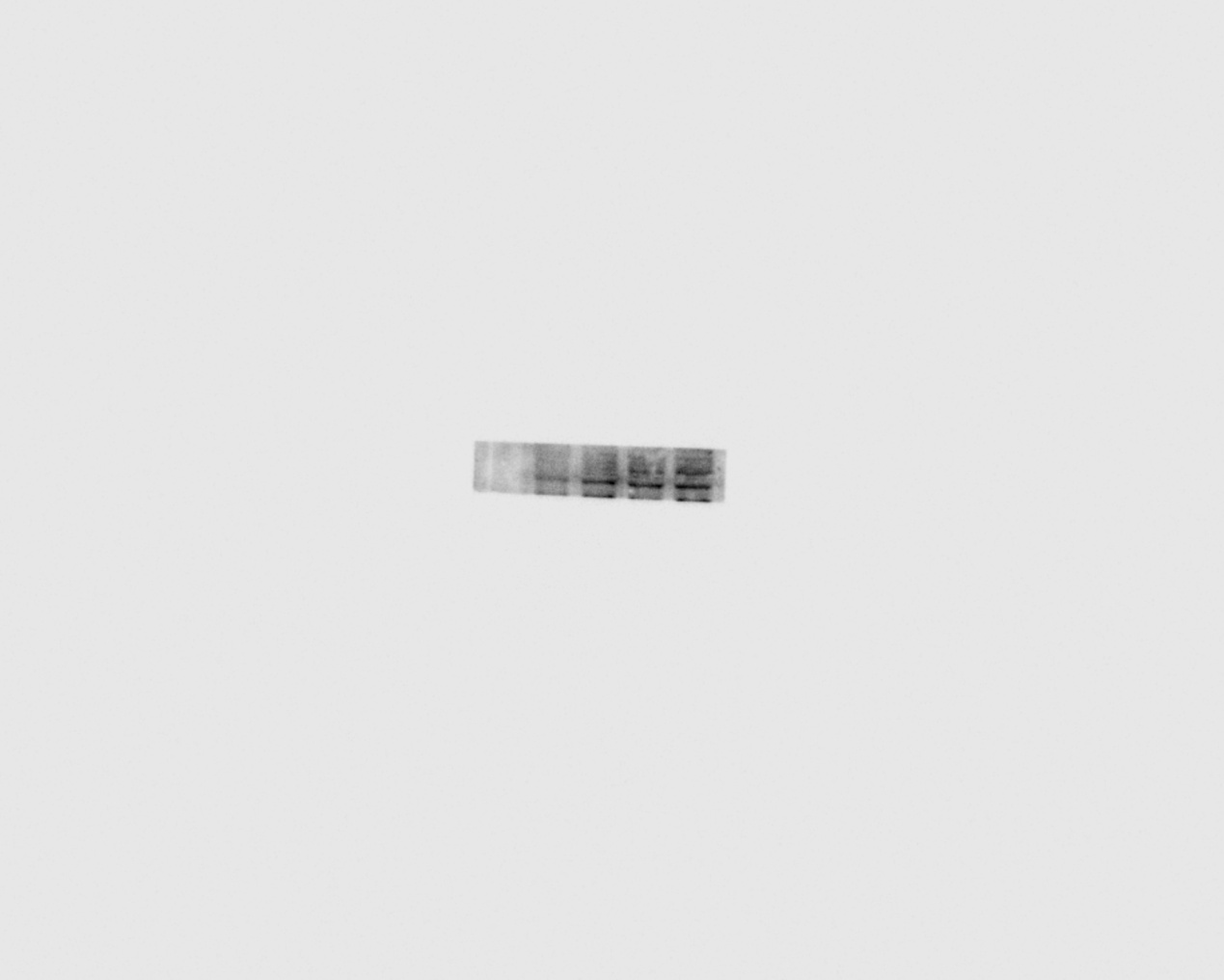


**HIF-1a**

IL-34(ng/ml): 0 25 50 100


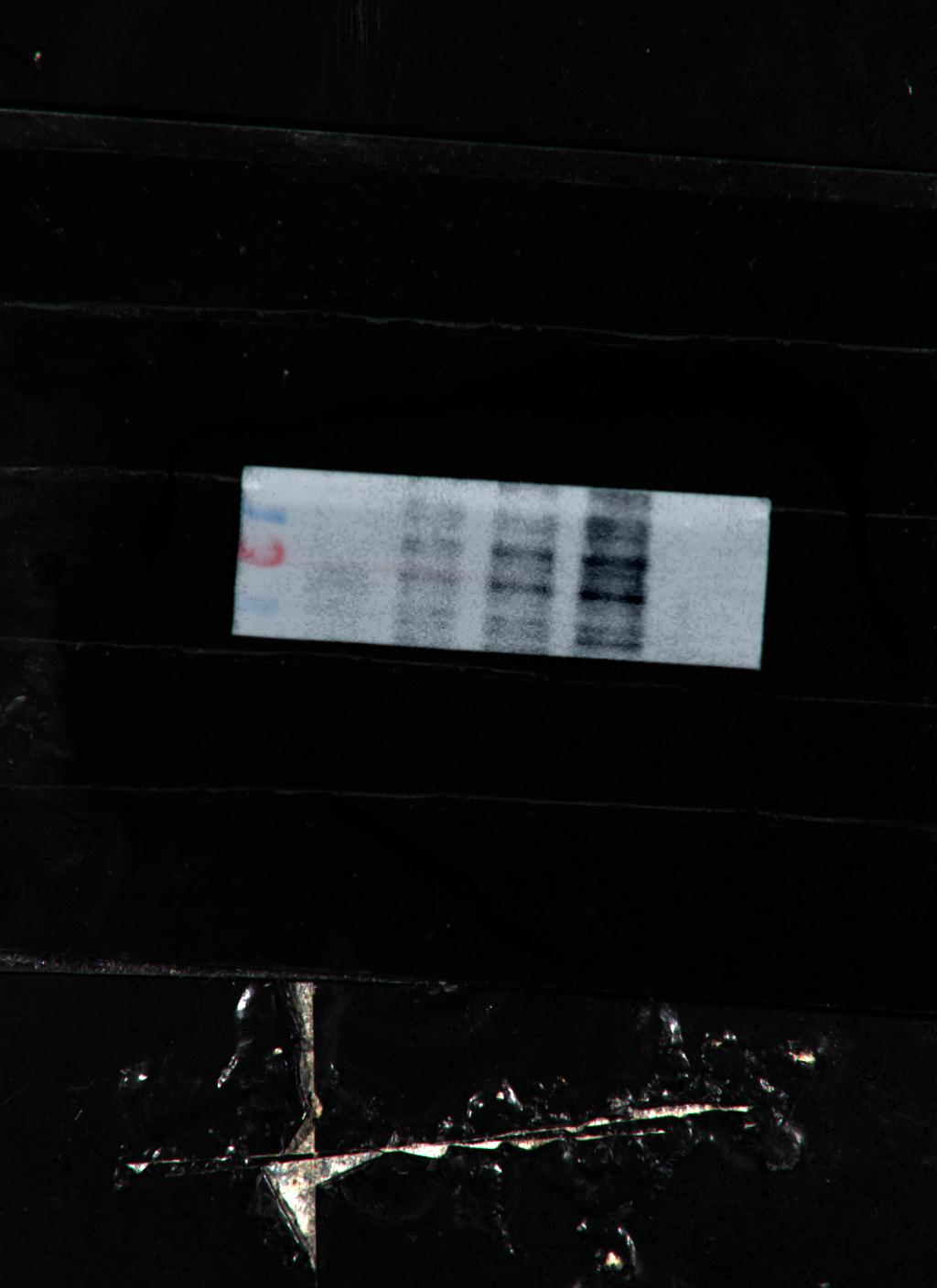


**VEGF**

IL-34(ng/ml): 0 25 50 100


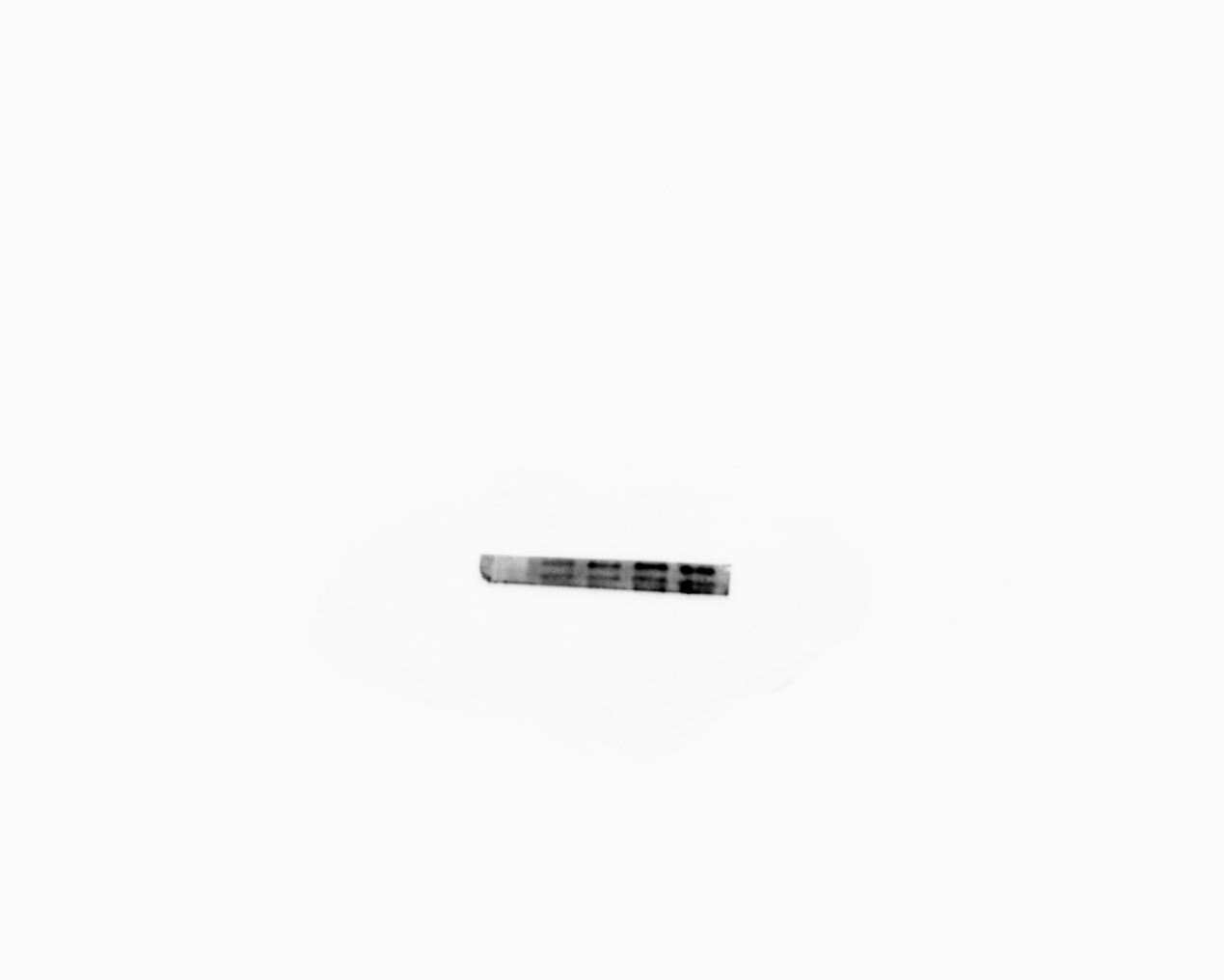


**VEGF**

IL-34(ng/ml): 0 25 50 100


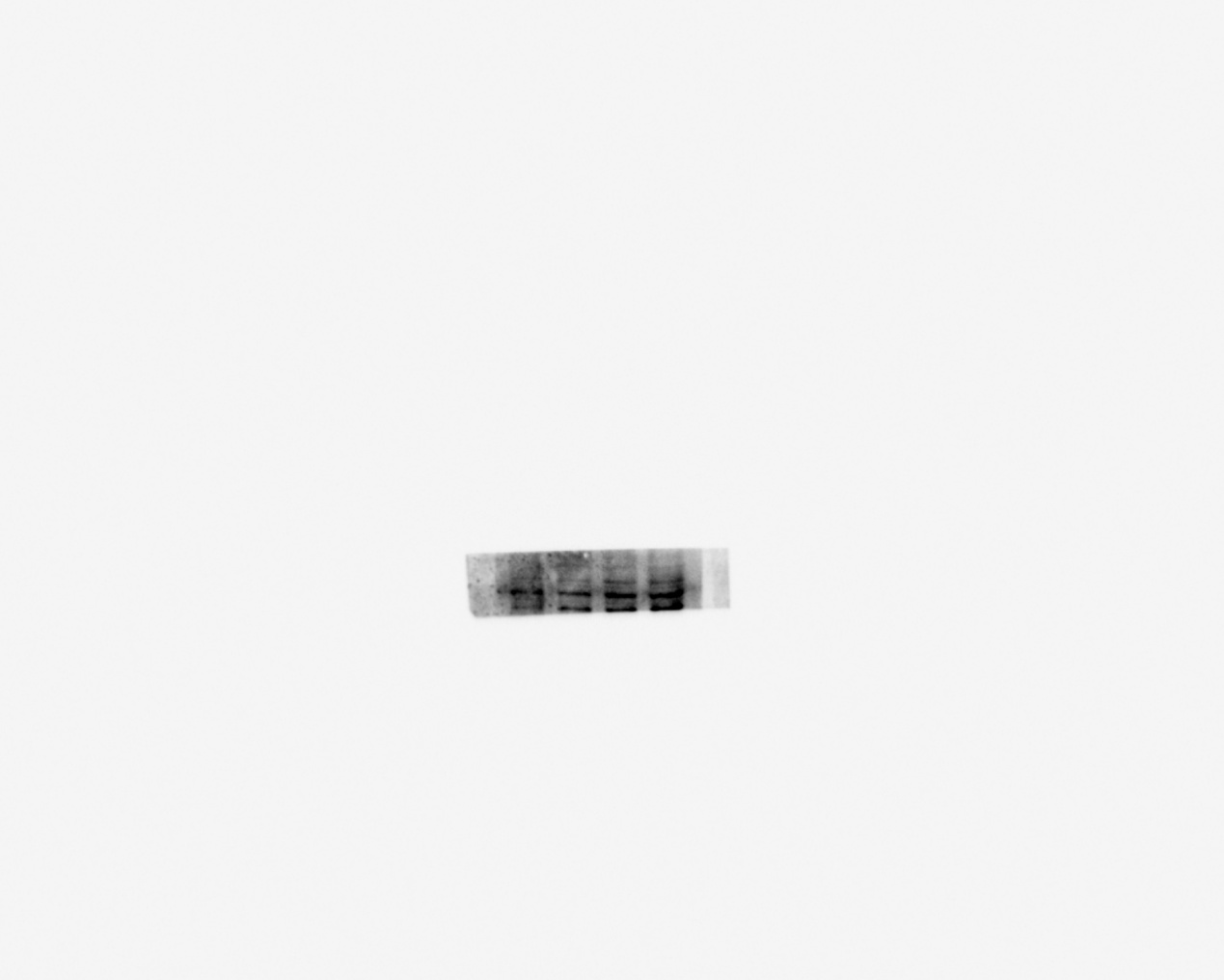


**IL-6**

IL-34(ng/ml): 0 25 50 100 100+PB


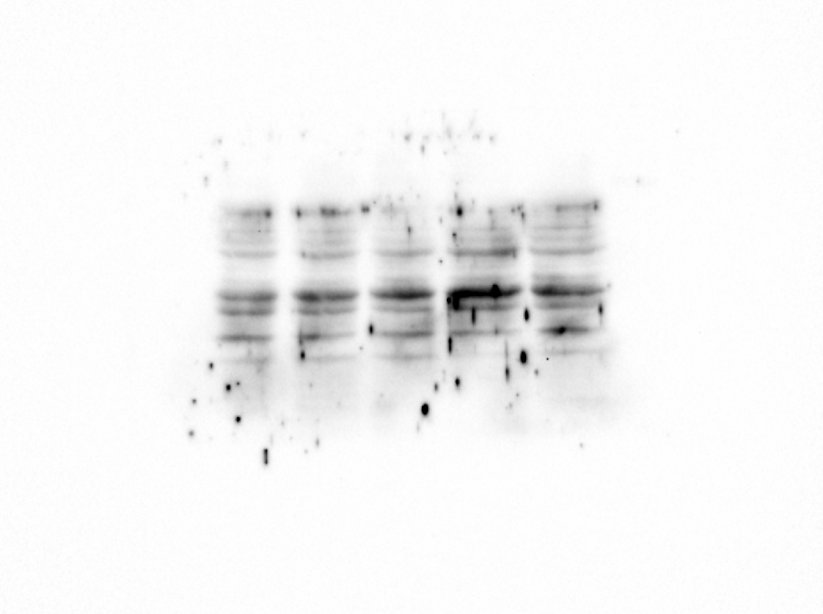


**IL-6**

IL-34(ng/ml): 0 25 50 100


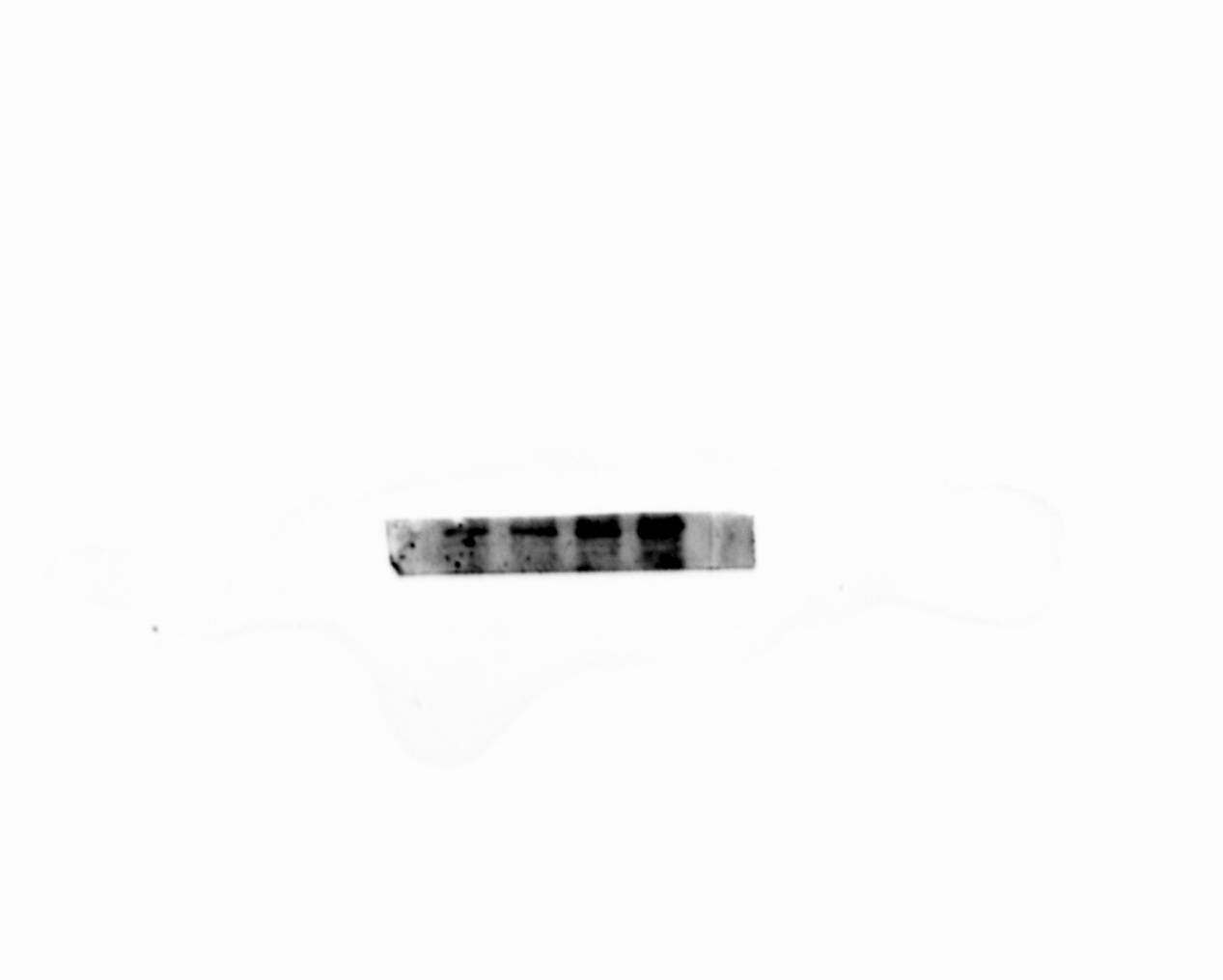


**TNF-a**

IL-34(ng/ml): 100 100+PB


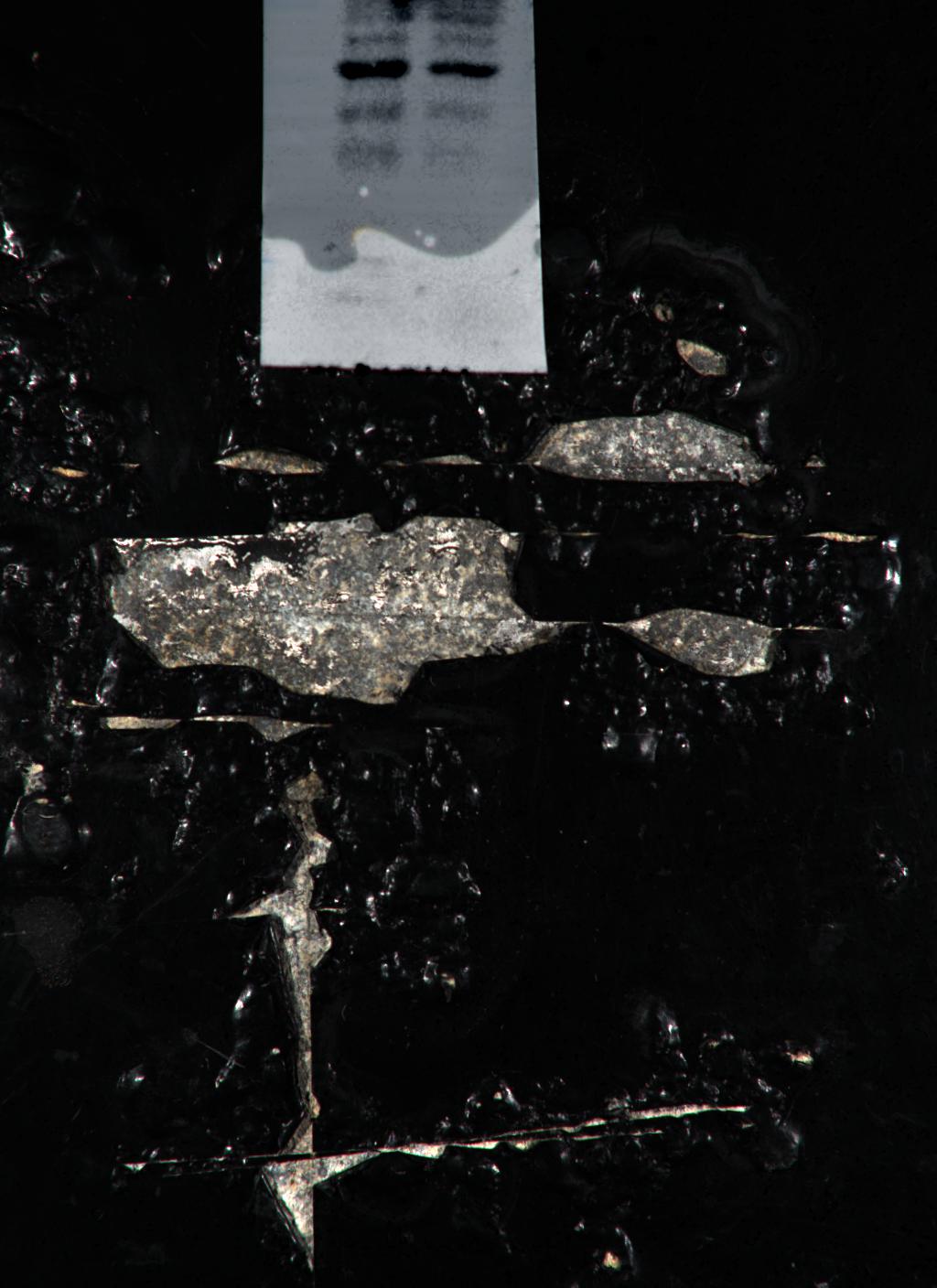


**TNF-a**

IL-34(ng/ml): 100 100+PB


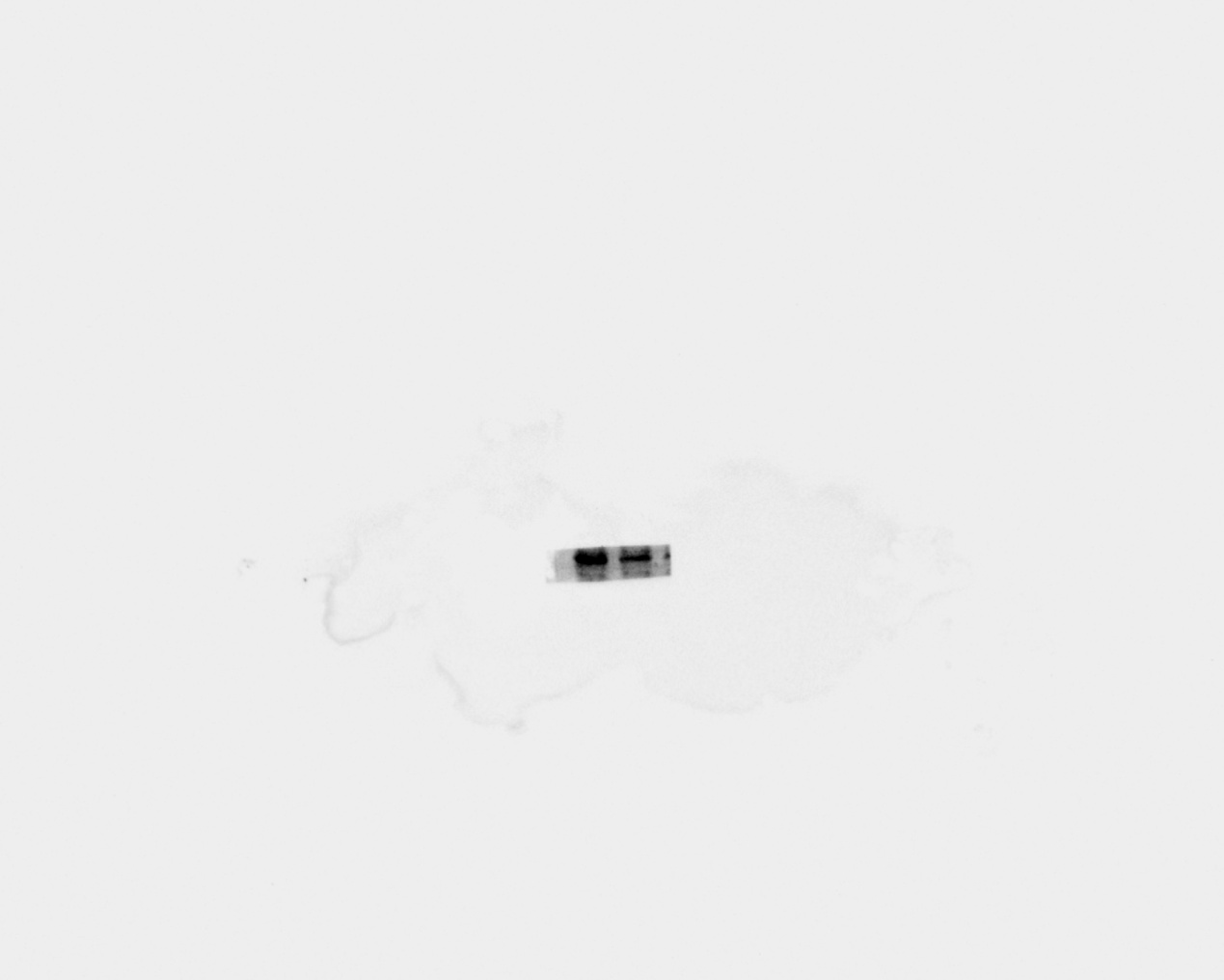


**IL-6**

IL-34(ng/ml): 100 100+PB


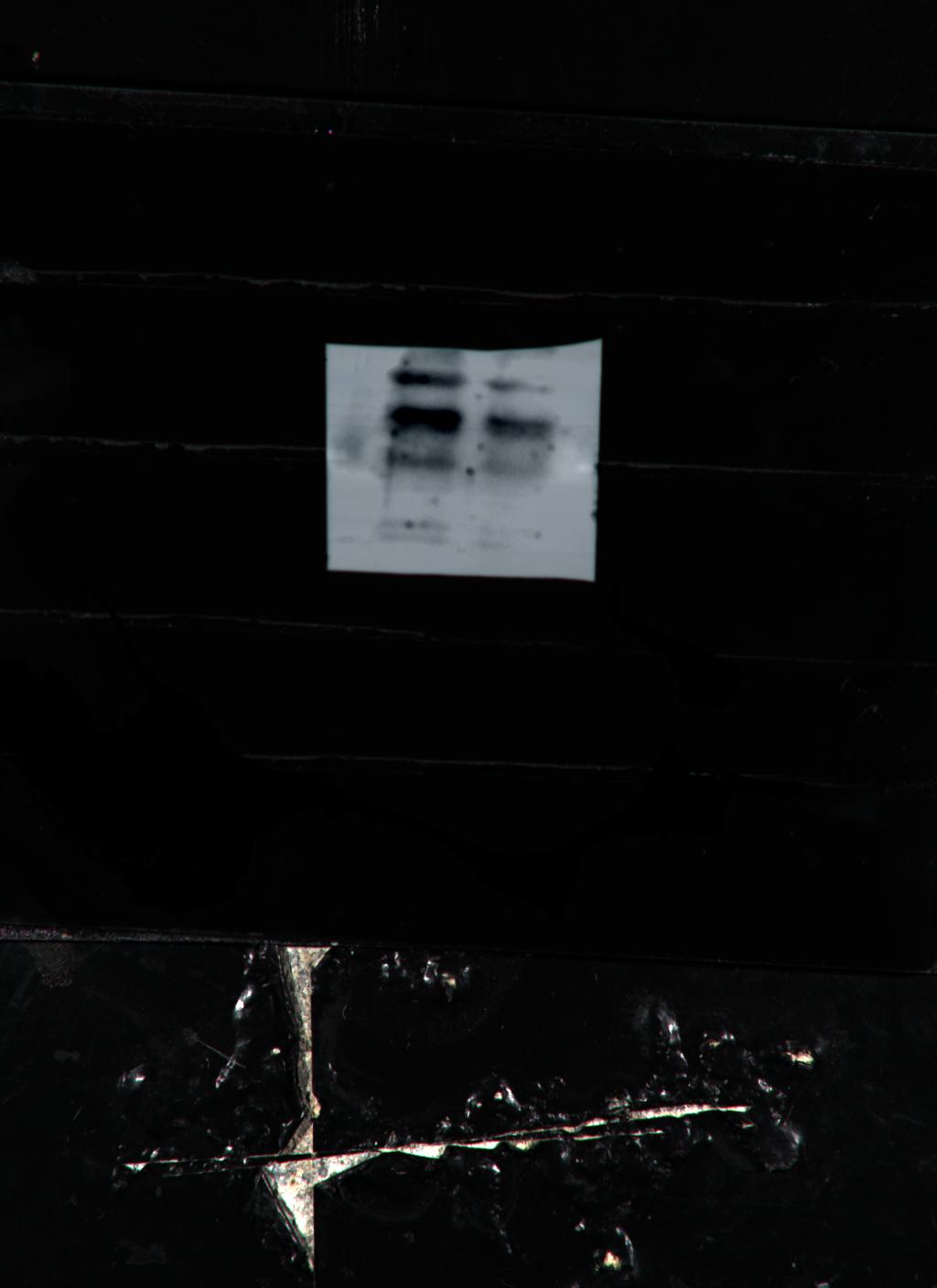


**IL-6**

IL-34(ng/ml): 100 100+PB


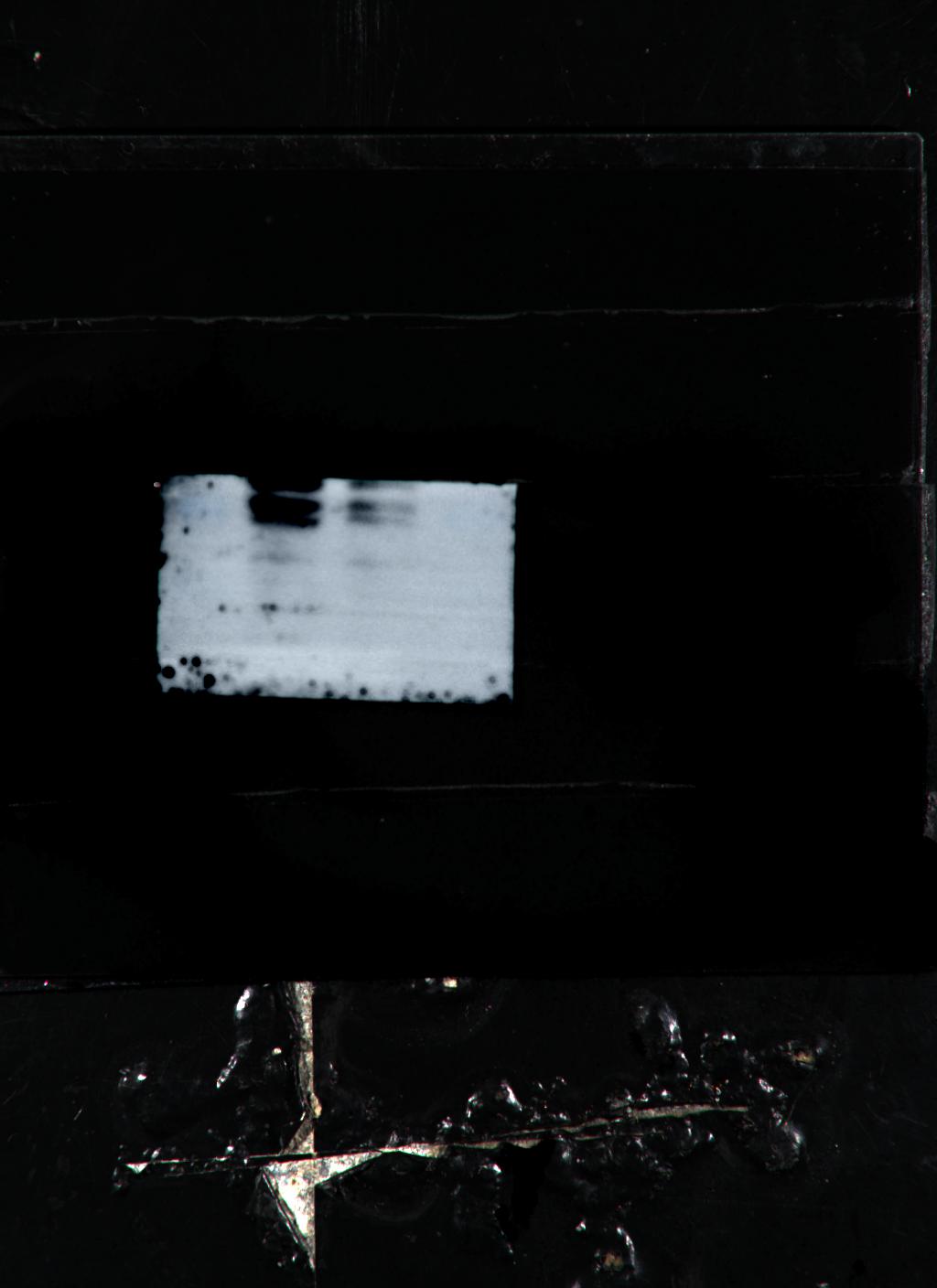


**IL-6**

IL-34(ng/ml): 100 100+PB


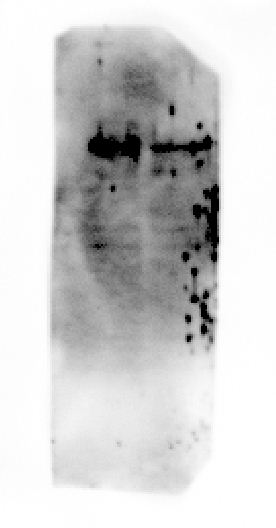


**VEGF**

IL-34(ng/ml): 100 100+PB


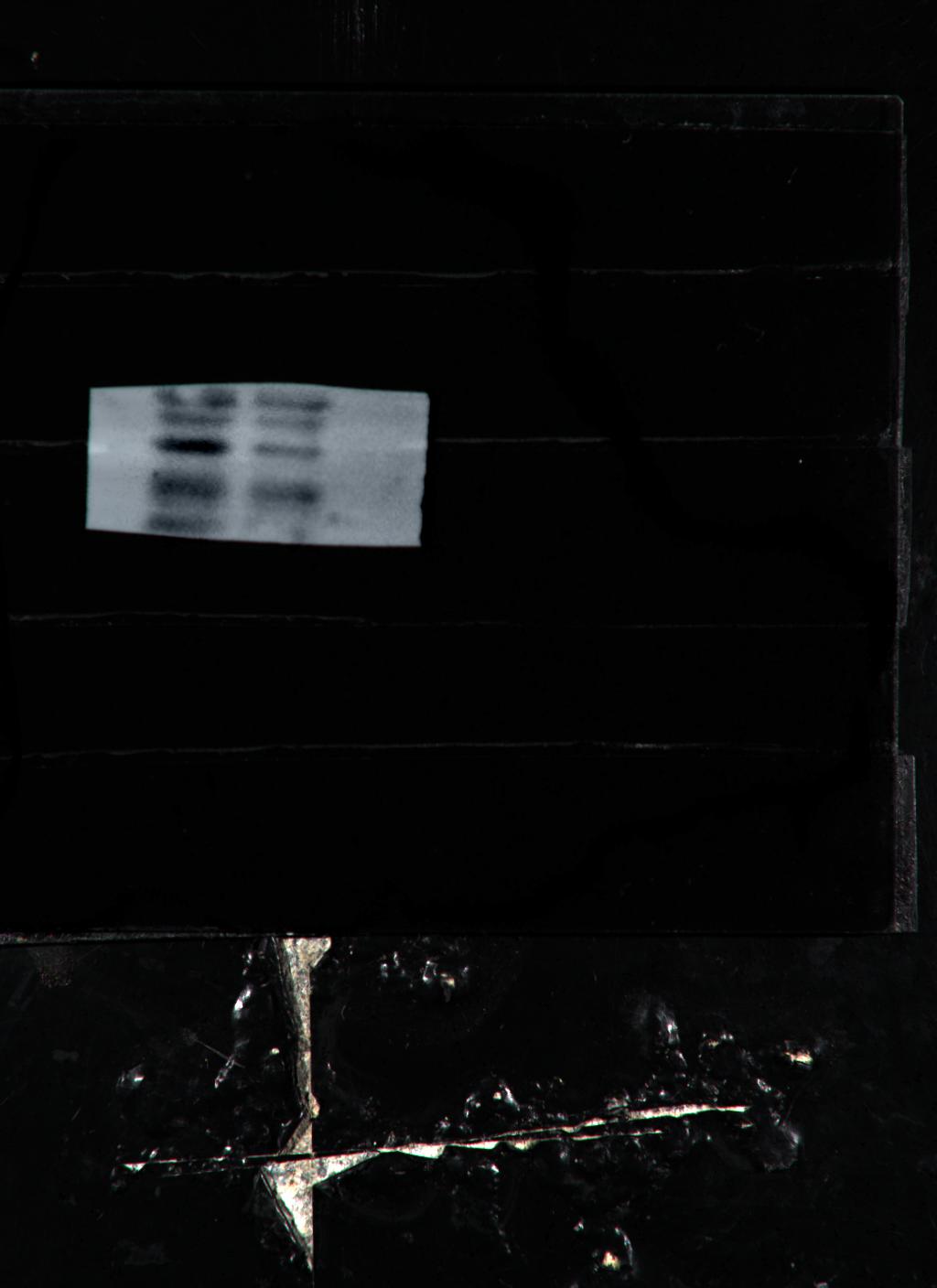


**VEGF**

IL-34(ng/ml): 100 100+PB


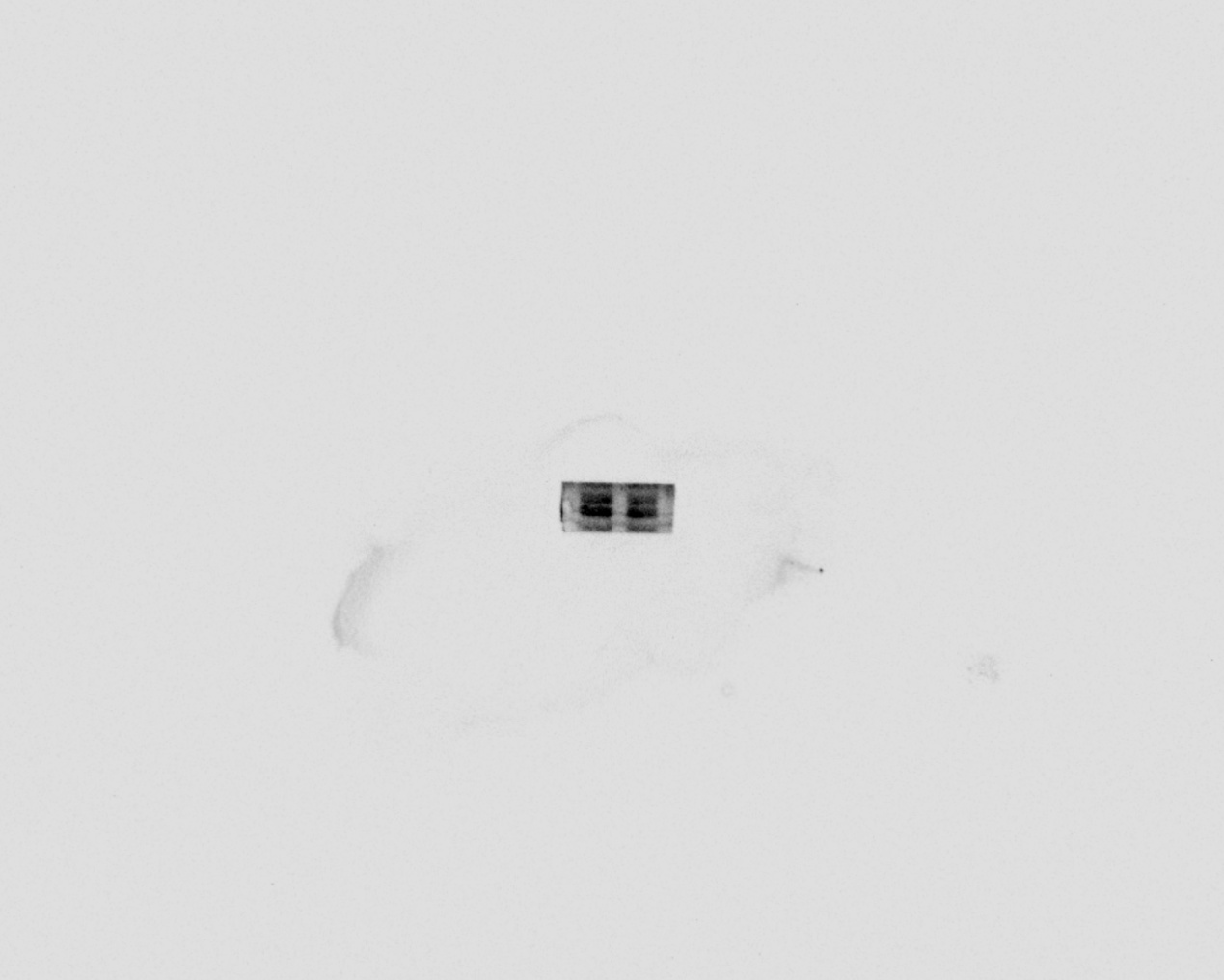


HIF-1α

IL-34(ng/ml): 100 100+PB


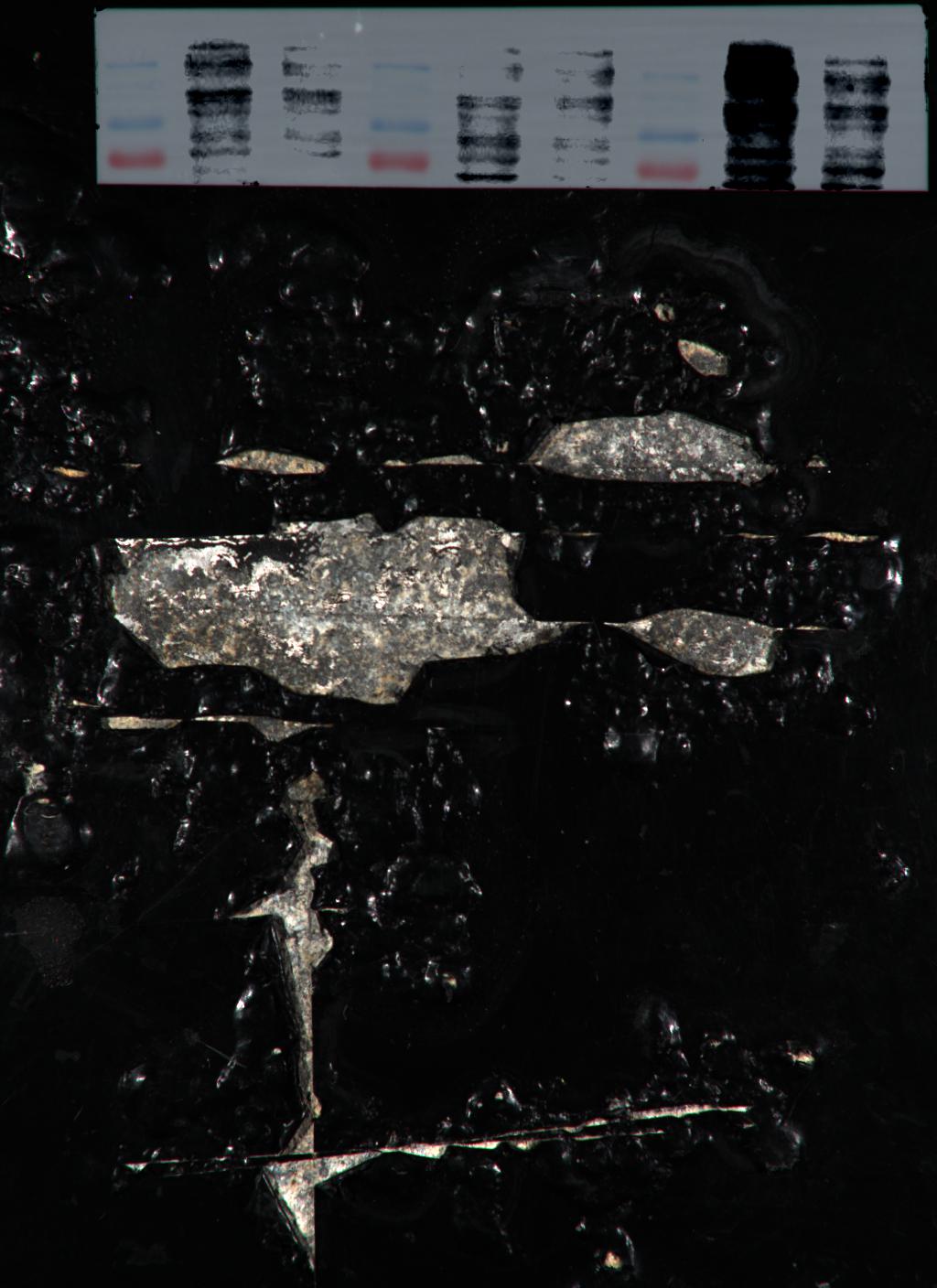


HIF-1α

IL-34(ng/ml): 100 100+PB


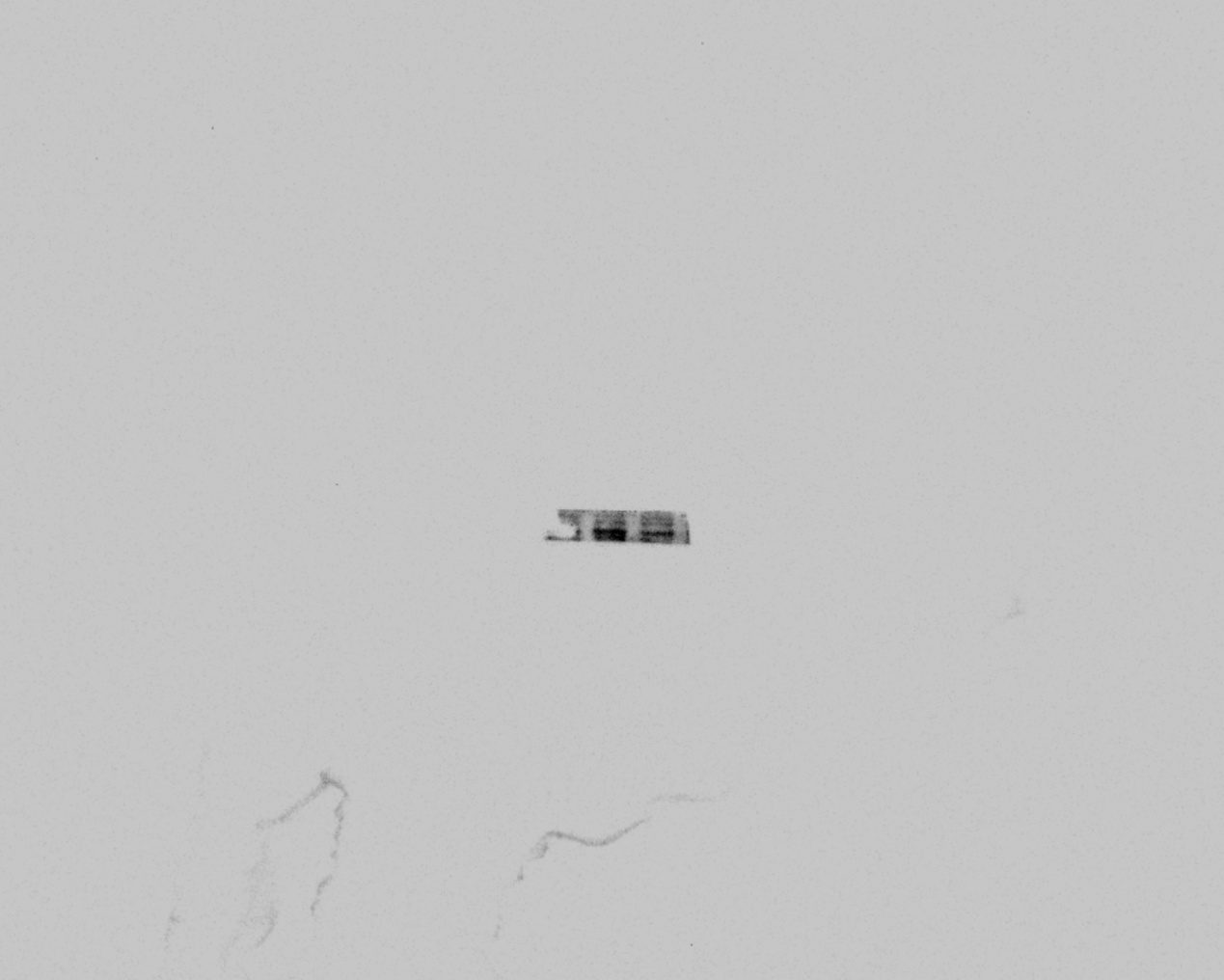


**GAPDH**

IL-34(ng/ml): 100 100+PB

**
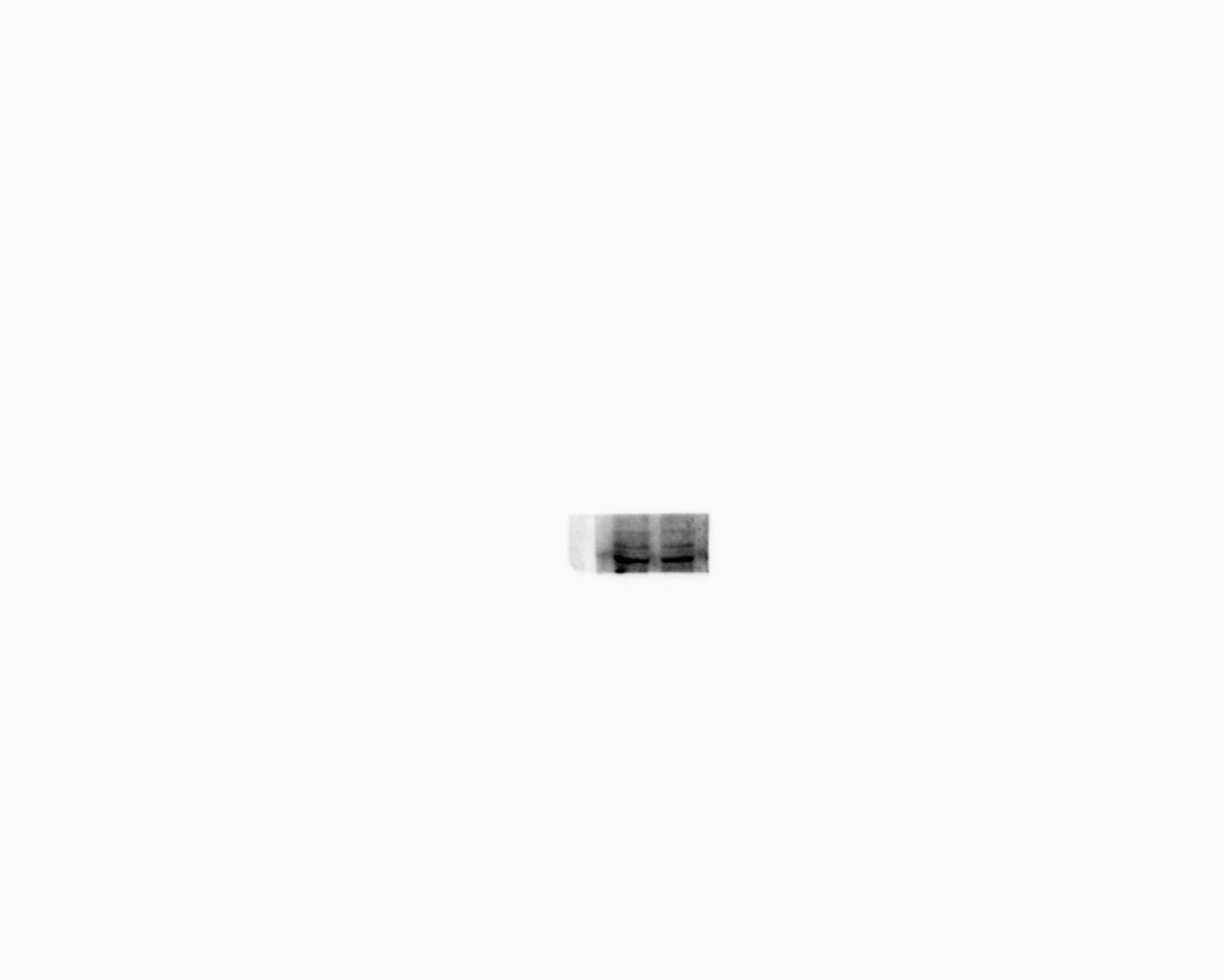
**

**GAPDH**

IL-34(ng/ml): 100 100+PB

**
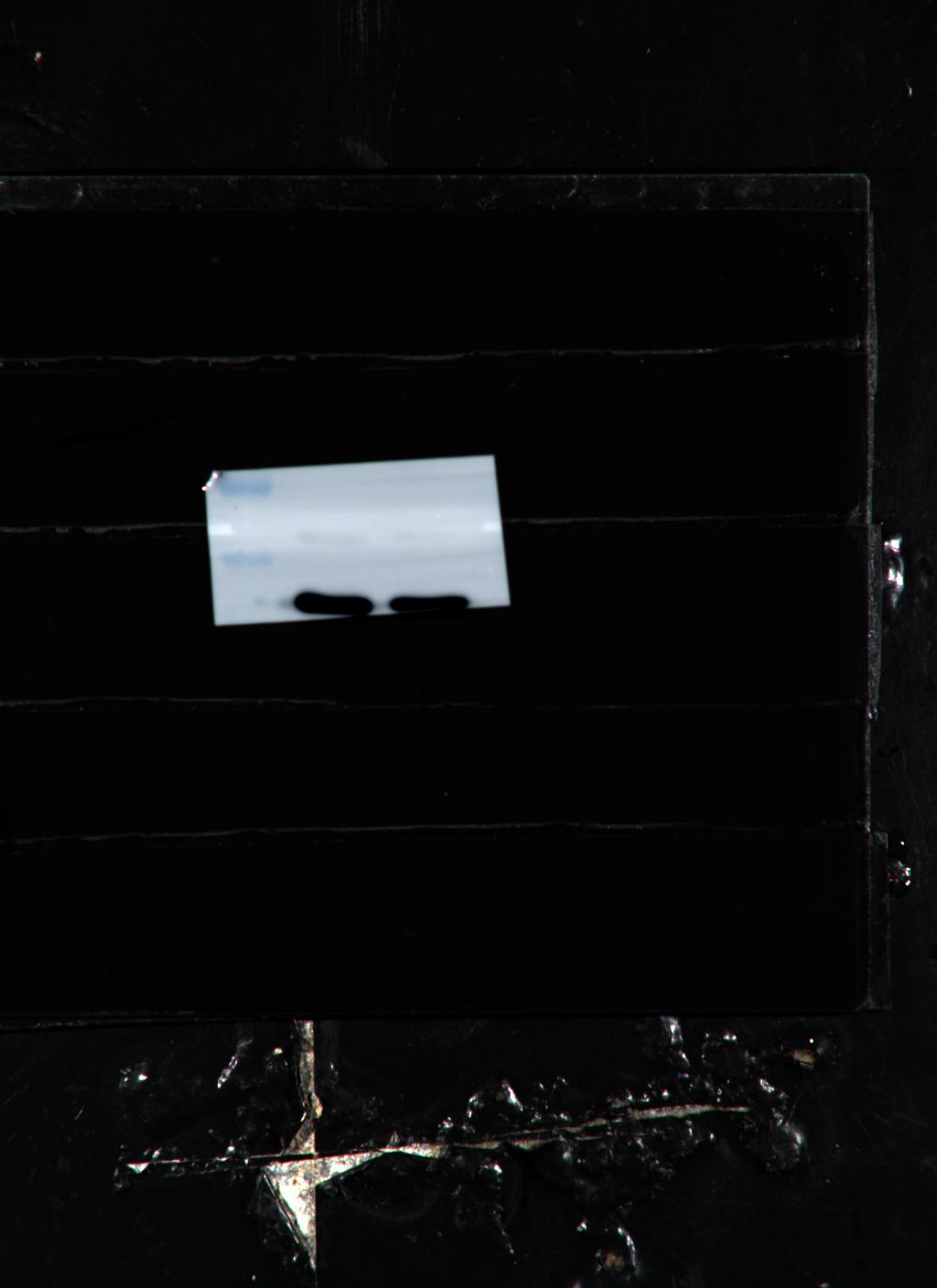
**
